# HmuY proteins of the *Porphyromonas* genus show diversity in heme-binding properties

**DOI:** 10.3389/fcimb.2025.1560779

**Published:** 2025-05-13

**Authors:** Michał Śmiga, Teresa Olczak

**Affiliations:** Laboratory of Medical Biology, Faculty of Biotechnology, University of Wrocław, Wrocław, Poland

**Keywords:** HmuY, hemophore-like protein, *Porphyromonas*, *Porphyromonas gingivalis*, virulence, evolution

## Abstract

**Introduction:**

Bacteria of the *Porphyromonas* genus, belonging to the Bacteroidota phylum, colonize various host niches in health and disease. As heme auxotrophs, they rely on heme uptake for iron and protoporphyrin IX. A key heme acquisition system in *Porphyromonas gingivalis* is the Hmu system, where the hemophore-like HmuY^Pg^ protein plays a major role. HmuY^Pg^ coordinates heme-iron using two histidines, whereas other known HmuY proteins produced by other Bacteroidota members prefer a pair of histidine-methionine or two methionines. Some of them bind heme *via* the protoporphyrin ring without heme-iron coordination, similar to the *P. gingivalis* HusA protein.

**Methods:**

This study used bioinformatics, spectroscopic, and electrophoretic methods to compare the genomic organization of the Hmu system and the structural and functional properties of HmuY proteins within the *Porphyromonas* genus.

**Results and Discussion:**

We revealed variations in the heme-binding properties of proteins belonging to the HmuY family and susceptibility to modifications in their heme-binding pockets. These findings suggest that HmuY proteins may have undergone evolutionary adaptations to enhance bacterial survival in the human microbiome, contributing to dysbiosis and disease development. These evolutionary changes may explain the superior heme-binding ability of *P. gingivalis* HmuY^Pg^ compared to HmuY homologs produced by other *Porphyromonas* species.

## 1 Introduction

Bacteria belonging to the Bacteroidota (formerly Bacteroidetes) phylum (Oren and Garrity, 2021) inhabit various host microbiomes, can spread in the host, and cause diseases (Acuna-Amador and Barloy-Hubler, 2020). Besides engagement in infections and inflammatory diseases, they are associated with systemic diseases and cancers (Aguayo et al., 2018; Mei et al., 2020; Baima et al., 2023). Among them is the *Porphyromonas* genus, which comprises ~25 species according to the List of Prokaryotic Names with Standing in Nomenclature (LPSN; https://lpsn.dsmz.de/) accessed on 10.01.2025 (Parte et al., 2020). *Porphyromonas* genus (Gibson and Genco, 2006; Summanen et al., 2015; Guilloux et al., 2021) comprises bacteria that inhabit the oral cavity, gastrointestinal tract, and urogenital tract of humans and animals (Collins et al., 1994; Love et al., 1994; Paster et al., 1994; Love, 1995; Finegold et al., 2004; Summanen et al., 2009; Sakamoto and Ohkuma, 2013; Sakamoto et al., 2015; Sato et al., 2015; Kim et al., 2016; Zamora-Cintas et al., 2018; Acuna-Amador and Barloy-Hubler, 2020; Cobo et al., 2021; Guilloux et al., 2021; Morales-Olavarria et al., 2023). Some of them, such as *Porphyromonas catoniae*, belong to anaerobic commensal flora (Keravec et al., 2019). Others, like *P. gingivalis*, belong to opportunistic pathogens that can be found in low numbers in the healthy oral cavity but in high numbers in patients with periodontitis (Ximenez-Fyvie et al., 2000; Gomes et al., 2005; Bik et al., 2010; Boyapati et al., 2024; Lamont and Kuboniwa, 2024). Importantly, some species, such as *Porphyromonas pogonae* human origin PP01–1 strain, harbor antimicrobial resistance genes (Huang et al., 2024), while others, like *P. gingivalis* isolates, are prone to acquiring antibiotic resistance (Kulik et al., 2019; Rams et al., 2023; Ng et al., 2024).

To proliferate and cause disease, pathogens must access nutrients, including iron and heme (Andrews et al., 2003). Iron plays a crucial role in all living organisms and, due to its redox activity, serves as a cofactor for metabolic enzymes, electron chain components, or DNA biosynthesis (Palmer and Skaar, 2016). Pathogens frequently utilize heme as a source of iron and protoporphyrin IX (PPIX). Heme is crucial for various cellular processes such as electron transfer, aerobic respiration, and gas sensing (Choby and Skaar, 2016). Both free iron and free heme are unavailable in the host because of their toxicity, resulting from the Fenton reaction of Fe(II), a mechanism generating reactive oxygen species involved in membrane protein and lipid oxidation and DNA damage (Koppenol and Hider, 2019). To overcome iron or heme toxicity, as well as to make iron and heme inaccessible for pathogens, both compounds are sequestered by host proteins as part of the innate immune response (Choby and Skaar, 2016; De Simone et al., 2023). Iron-sequestering proteins such as transferrin in the serum and lactoferrin in mucous secretions (Cherayil, 2011; Sheldon et al., 2016) limit free iron concentration to about 10^-18^ M (Bullen et al., 1978; Cherayil, 2011; Sheldon et al., 2016) which is far below the levels required to support bacterial growth (10^-8^-10^-6^ M) (Guerinot, 1994). Also, the concentration of free heme in the serum is at a negligible level (Khan and Quigley, 2011) due to the production of heme-sequestering proteins: albumin and hemopexin (Chiabrando et al., 2014).

Therefore, pathogens evolved mechanisms to overcome host iron- and heme-limiting strategies, including utilization of iron-chelating siderophores, direct binding of host iron- or heme-binding proteins to bacterial receptors, or utilization of heme-sequestering hemophores (Palmer and Skaar, 2016; Wandersman and Delepelaire, 2004; Sheldon et al., 2016; Donegan, 2022; Olczak et al., 2024). A typical heme uptake system of Gram-negative bacteria is the Hmu system of *Yersinia pestis* (HmuRSTUV) (Hornung et al., 1996; Thompson et al., 1999) or the Hem system of *Yersinia enterocolitica* (Stojiljkovic and Hantke, 1992). Heme is transported from the external environment across the outer membrane through the TonB-dependent outer membrane receptor (TDR) (e.g., *Y. pestis* HemR or *Y. enterocolitica* HmuR) (Contreras et al., 2014; Silale and van den Berg, 2023). Depending on the bacterium, TDRs recognize hemoglobin, haptoglobin-hemoglobin, or heme released from hemoproteins and then transport heme across the outer membrane into the periplasmic space (Wyckoff et al., 1998). Some bacteria utilize accessory heme-binding proteins, such as HasA hemophores from *Serratia marcescens* (Arnoux et al., 1999) or *Yersinia pestis* (Kumar et al., 2013). Hemophores bind free heme or sequester it from host hemoproteins and deliver it to TDRs, thus facilitating heme acquisition. In the periplasmic space heme is shuttled by periplasmic binding proteins (*e.g.*, *Y. pestis* HmuT or *Y. enterocolitica* HemT) and transported into the cytoplasm by inner membrane ABC transporters (*e.g*., *Y. pestis* HmuU and HmuV or *Y. enterocolitica* HemU and HemV) (Wyckoff et al., 1998; Ho et al., 2007; Chu and Vogel, 2011). In the cytoplasm, proteins such as HmuS and HemS transfer heme to enzymes that either utilize heme or break heme down to release iron from heme (*e.g*., heme oxygenases) (Lansky et al., 2006, Schneider and Paoli, 2005).

Among the best-characterized heme acquisition mechanisms identified in members of the Bacteroidota phylum is the *P. gingivalis* Hmu system (HmuYRSTUV) (Wojtowicz et al., 2009a, 2009b; Bielecki et al., 2018, 2020; Sieminska et al., 2021; Antonyuk et al., 2023; Śmiga and Olczak, 2024). The first protein encoded on the *P. gingivalis hmu* operon, HmuY^Pg^, is the first representative of the novel HmuY family comprising hemophore-like proteins, different from classical HasA hemophores or *P. gingivalis* HusA^Pg^ hemophore-like protein (Olczak et al., 2024). HmuR^Pg^ is a typical TDR involved in the transport of heme through the outer membrane (Smalley and Olczak, 2017; Olczak et al., 2024). HmuS is a putative reverse ferrochelatase (homologous to *Bacteroides fragilis* BtuS2 protein) which may remove iron from heme (our unpublished data). HmuT and HmuU proteins show homology to ATPase and MotA/TolQ/ExbB proton channel family proteins, respectively, while HmuV shows no homology to any known protein family. HmuTUV proteins are most likely involved in further heme, PPIX, or iron transport into the bacterial cell (Smalley and Olczak, 2017; Rocha et al., 2019).

HmuY homologs differ in the type of amino acids involved in heme-iron coordination, which results in different heme-binding capacities depending on the heme-iron redox state. So far, characterized HmuY proteins coordinating heme iron by two methionines bind heme preferentially under reducing conditions (Bielecki et al., 2018, 2020; Sieminska et al., 2021; Antonyuk et al., 2023). Among the best-characterized are *P. intermedia* HmuY^Pi-1^ (formerly PinO), *P. intermedia* HmuY^Pi-2^ (formerly PinA), *T. forsythia* HmuY^Tf^ (formerly Tfo), *Bacteroides vulgatus* HmuY^Bv^ (formerly Bvu), and *B. fragilis* HmuY^Bf-1^ (formerly BfrA). In contrast, *P. gingivalis* HmuY^Pg^ coordinates heme iron by two histidines, which results in a high affinity of heme binding in both oxidized and reduced environments (Wojtowicz et al., 2009a, 2009b). HmuY homolog from *Porphyromonas endodontalis* (HmuY^Pe^) uses a histidine-methionine pair to coordinate heme-iron (Śmiga and Olczak, 2024). In addition to these main amino acids involved in hemophore-like proteins in the coordination of heme-iron, crystallographic data have shown that other amino acid residues, including arginine and tyrosine, play a supporting role in heme binding by HmuY proteins (Wojtowicz et al., 2009b; Antonyuk et al., 2023). HmuY proteins also differ in the ligands they bind: the second *B. fragilis* HmuY homolog, HmuY^Bf-2^ (formerly BfrB), binds heme but most likely by interacting with the side groups of PPIX without heme-iron coordination, whereas the third *B. fragilis* homolog, HmuY^Bf-3^ (formerly BfrC), binds neither heme nor PPIX (Antonyuk et al., 2023).

In addition to the HmuY protein, *P. gingivalis* produces another hemophore-like protein, HusA^Pg^, that is not assigned to the HmuY family; however, similarly to HmuY^Bf-2^, it prefers binding metal-free porphyrins (Gao et al., 2018; Śmiga et al., 2023a, 2024). HusA^Pg^ is a part of the Hus system encoded on the *hus* operon, comprising genes encoding HusB, a typical TDR, and HusC and HusD proteins whose functions are unknown (Gao et al., 2010, 2018).

This study aimed to perform theoretical and experimental analyses of the Hmu systems identified in the *Porphyromonas* genus, with a particular emphasis on HmuY proteins, to assess their characteristics and potential role in heme acquisition. We compared them with the well-characterized *P. gingivalis* Hmu system proteins and systems identified in other selected Bacteroidota members to elucidate their function in the *Porphyromonas* species and explore interspecies variations. We hypothesize that HmuY proteins may have undergone evolutionary adaptations to enhance bacterial survival in the human microbiome, contributing to dysbiosis and disease development. These evolutionary changes may explain the superior heme-binding ability of *P. gingivalis* HmuY^Pg^ compared to HmuY homologs produced by other *Porphyromonas* species.

## 2 Materials and methods

### 2.1 Gene and protein sequence acquisition

All sequences were obtained from the GenBank database (https://www.ncbi.nlm.nih.gov/genbank/) or the Protein database (https://www.ncbi.nlm.nih.gov/protein). The search for protein homologs was performed using BLASTP and PSI-BLAST (https://blast.ncbi.nlm.nih.gov/Blast.cgi) based on proteins identified in the *P. gingivalis* W83 strain. The sequences were selected manually and protein homologs that contained at least 50% of sequence coverage were used for further analyses. The sequences were chosen from selected strains listed in **Supplementary Table S1**.

### 2.2 Phylogenetic analysis

Phylogenetic trees were created by neighbor-joining comparison of nucleotide sequences of genes encoding *16S rRNA* or amino acid sequences of selected proteins with the Multiple Sequence Alignment tool – Clustal Omega (Madeira et al., 2022). For phylogenetic trees based on amino acid sequences, data were analyzed using Simple Phylogeny (Madeira et al., 2022) with Clustal Tree format and distance correction. Phylogenetic trees were visualized using iTOL (Letunic and Bork, 2021).

### 2.3 Operon prediction

The prediction of operons and gene clusters was performed using an Operon mapper (https://biocomputo.ibt.unam.mx/operon_mapper/).

### 2.4 Prediction of amino acids involved in ligand binding

Protein sequences were compared using the Clustal Omega tool (Madeira et al., 2022) and visualized using Jalview (Waterhouse et al., 2009). Three-dimensional protein structures were obtained from the RCSB Protein Data Bank (PDB, https://www.rcsb.org) (**Supplementary Table S1**). Theoretical three-dimensional structures were obtained from the AlphaFold Protein Structure Database (https://alphafold.com) (Jumper et al., 2021; Varadi et al., 2022) or predicted with Phyre2 (Kelley et al., 2015) and I-TASSER (Yang and Zhang, 2015). Predicted HmuY^Pg^, HmuY^Pe^, and HmuY^Tf^ site-directed mutagenesis variants protein models were additionally refined using ModRefiner (Xu and Zhang, 2011). All protein structures were visualized with UCSF Chimera (Pettersen et al., 2004).

### 2.5 Protein overexpression and purification

HmuY^Pg^, HusA^Pg^, HmuY^Tf^, and HmuY^Bf-2^ proteins were overexpressed and purified as described previously (Śmiga et al., 2023a; Ślęzak et al., 2020; Antonyuk et al., 2023) using plasmids listed in **Supplementary Table S2**. Briefly, proteins without the predicted signal peptide for HusA^Pg^ (MKTFKRIALLLVAGFAGLCATSA), HmuY^Tf^ (MKMRNVMTLALVALSLAFVGC), HmuY^Bf-2^ (MNNKNKFRFAILLFGVLSAFIITAC), or signal peptide with an additional five amino acids in the case of HmuY^Pg^ (MKKIIFSALCALPLIVSLTSCGKKK) but containing 8×His-MBP (Maltose binding protein) tag and amino acids recognized by Factor Xa at the N-terminus were overexpressed using *Escherichia coli* BL21CodonPlus (DE3)-RIL cells (Agilent Technologies, Santa Clara, CA, USA). Proteins were purified from the soluble fraction of the *E. coli* cell lysates using amylose resin (New England Biolabs). The 8×His-MBP tag was cut off with Factor Xa (New England Biolabs) and removed using TALON resin (Sigma-Aldrich).

HmuY^Pe^ was overexpressed and purified as described previously (Śmiga and Olczak, 2024) using the plasmid listed in **Supplementary Table S2**. Briefly, HmuY^Pe^ without the predicted signal peptide (MVLGVASCRP) but with a 6×His tag and amino acids recognized by Factor Xa at the N-terminus of the protein was overexpressed using *E. coli* BL21CodonPlus (DE3)-RIL cells (Agilent Technologies). Protein was purified from the soluble fraction of the *E. coli* cell lysates using TALON resin (Sigma-Aldrich), and the 6×His tag was cut off with Factor Xa (New England Biolabs). To remove the 6×His tag and the uncleaved 6×His tag-containing protein, nickel-immobilized resin (Ni-NTA; New England Biolabs) was used.

Protein concentration was measured using spectrophotometric method and empirical molar absorption coefficients: HmuY^Pg^ (ϵ_280_ = 36.86 mM^−1^ cm^−1^) (Wojtowicz et al., 2009a), HmuY^Pe^ (ϵ_280_ = 35.56 mM^−1^ cm^−1^) (Śmiga and Olczak, 2024), HmuY^Tf^ (ϵ_280_ = 26.32 mM^−1^ cm^−1^) (Bielecki et al., 2018), HmuY^Bf-2^ (ϵ_280_ = 25.08 mM^−1^ cm^−1^) (Antonyuk et al., 2023), and HusA^Pg^ (ϵ_280_ = 33.81 mM^−1^ cm^−1^) (Śmiga et al., 2023a).

To generate plasmids encoding variants of HmuY^Pe^ (H128M, M163H, and H128M/M163H), HmuY^Tf^ (M145H, M171H, and M145H/M171H), and HmuY^Bf-2^ (C153A), a QuikChange II XL Site-Directed Mutagenesis Kit (Agilent Technologies) and primers listed in **Supplementary Table S3** were used. Site-directed mutagenesis variants of HmuY^Pe^, HmuY^Tf^, HmuY^Pg^, and HmuY^Bf-2^ listed in **Supplementary Table S2** were overexpressed and purified as described above for unmodified proteins.

### 2.6 Preparation of porphyrin solutions

Porphyrin solutions were prepared as reported previously (Śmiga and Olczak, 2024; Antonyuk et al., 2023; Morgan and Muller-Eberhard, 1972). Briefly, heme (hemin chloride; Pol-Aura, Morąg, Poland) and mesoheme (iron-mesoporphyrin IX, FeMPIX; Sigma-Aldrich, St. Louis, MO, USA) solutions were prepared in 0.1 M NaOH, whereas protoporphyrin IX (PPIX; Fluka, Munich, Germany) and mesoporphyrin IX (MPIX; Sigma-Aldrich) solutions were prepared in DMSO. To determine heme and PPIX concentration, the empirical molar absorption coefficients ϵ_385_ = 58.5 mM^−1^ cm^−1^ and ϵ_405_ = 150 mM^−1^ cm^−1^ were used, respectively. FeMPIX and MPIX concentrations were calculated from the weighted portion of the porphyrin.

### 2.7 Characterization of porphyrin binding using spectroscopic methods

5 µM protein solution prepared in 20 mM sodium phosphate buffer, pH 7.4, containing 140 mM NaCl (PBS) was mixed with an equimolar concentration of heme, PPIX, FeMPIX, or MPIX. To determine the binding preference of iron-porphyrin or metal-free porphyrin, 5 µM protein was mixed with 5 µM heme and 5 µM PPIX or 5 µM FeMPIX and 5 µM MPIX. The UV-visible absorbance spectra of protein-porphyrin complexes were recorded using a double-beam Jasco V-750 spectrophotometer (Jasco GmbH, Pfungstadt, Germany) after 20 minutes of incubation at room temperature. The reduced conditions were formed by the addition of sodium dithionite (Sigma-Aldrich) to the final 10 mM concentration and mineral oil overlay of the sample (Sigma-Aldrich). Difference spectra were prepared by subtracting the spectrum of porphyrin alone from the spectrum of the protein-porphyrin complex. All analyses were performed in triplicate.

### 2.8 Detection of protein-porphyrin complexes using polyacrylamide gel electrophoresis

An alternative method based on PAGE was developed to detect protein-porphyrin complexes. Briefly, 10 µM protein with equimolar concentration of heme and/or PPIX were mixed, and the samples were incubated at 37°C for 30 minutes. 30 µl of the sample was mixed with 10 µl of the loading buffer composed of 0.4 M Tris/HCl buffer, pH 6.8, supplemented with 40% glycerol and 0.08% bromophenol blue. Then, 25 µl of the sample was separated on the 13.5% PAGE-separating gel prepared without sodium dodecyl sulfate (SDS), using standard electrode buffer with SDS. After electrophoresis, the PPIX-protein complexes were detected using the ChemiDoc Imaging System (Bio-Rad Laboratories, Hercules, CA, USA) and visualized with settings for Epi-far red fluorescence. To demonstrate heme-protein complexes, heme pseudoperoxidase activity using chemiluminescence staining was visualized. For this purpose, gels were incubated for 10 minutes in 15 ml of 3 times diluted oxidizing reagent (Perkin Elmer, Waltham, MA, USA). Then, 5 ml of enhanced luminol reagent (Perkin Elmer) was added and mixed briefly, and chemiluminescence was detected with the ChemiDoc Imaging System. The suitability of these methods for detecting the porphyrin compounds used in this study is shown in **Supplementary Figure S1**. To demonstrate equal loading of proteins, sodium dodecyl sulfate (SDS)-PAGE was performed using samples thermally denatured at 95°C for 10 minutes. After electrophoresis, proteins were stained with Coomassie Brilliant Blue G-250 (CBB-G250). All analyses were performed in triplicate.

## 3 Results

### 3.1 The main heme uptake systems in the *Porphyromonas* genus exhibit differences in their organization

**Figure 1:**
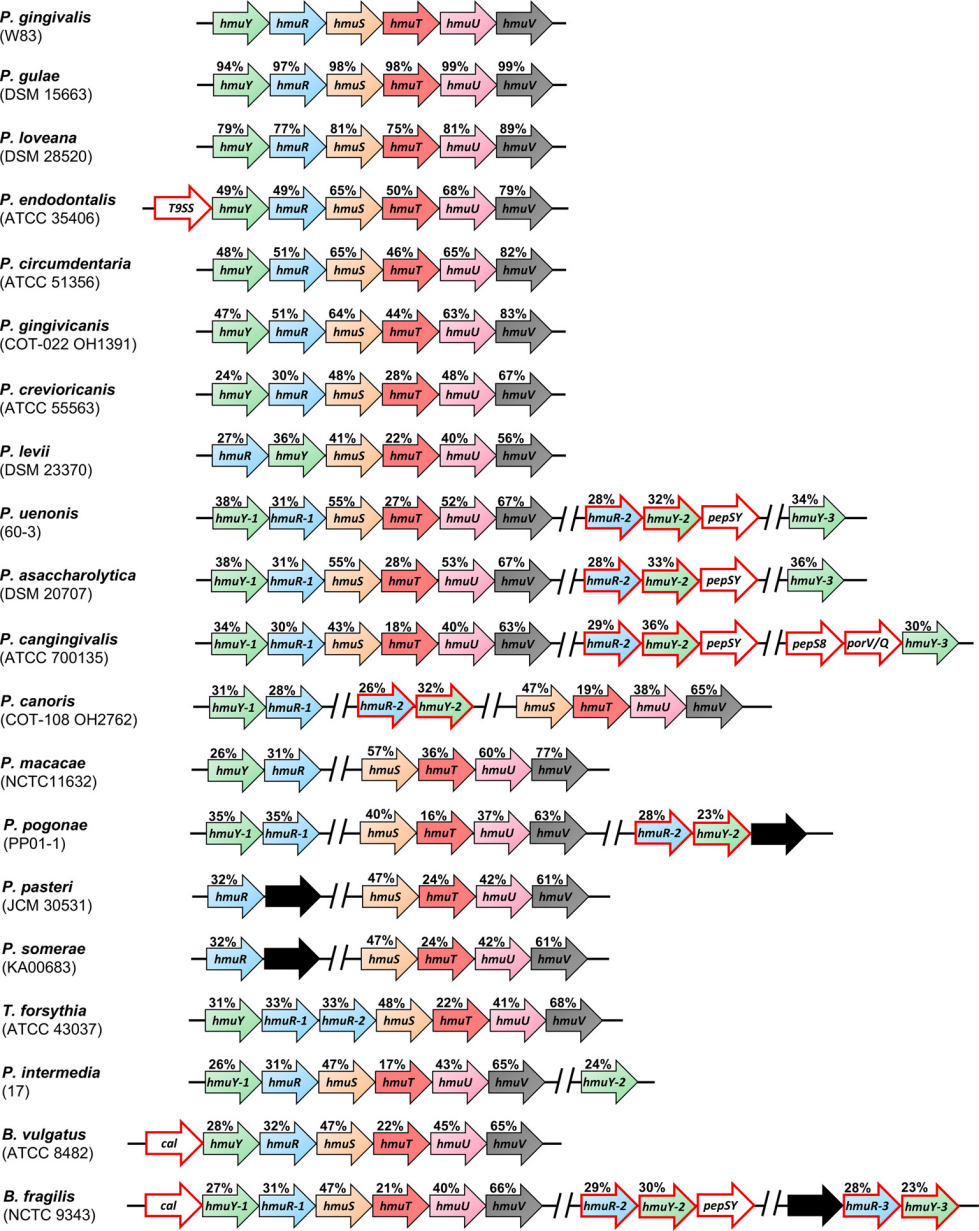
Schematic presentation of the organization of the operons or gene clusters encoding proteins of the Hmu system found in selected *Porphyromonas* species and other representative members of the Bacteroidota phylum. Arrows with a red outline represent genes not found in the *Porphyromonas gingivalis hmu* operon or genes encoding homologous proteins to those of the *P. gingivalis hmu* operon, but most likely exhibiting different functions. Black arrows indicate genes encoding hypothetical proteins or proteins of unknown function. *Prevotella intermedia hmu* operon and *hmuY-2* gene are located on chromosomes II and I, respectively. A gap between genes marked by // indicates that the gene clusters are in different genome regions. The values above the genes indicate the identity of the amino acid sequences with proteins from *P. gingivalis*. Strain names are indicated in brackets. *T9SS* – encodes a protein that contains T9SS type A sorting domain; *pepSY* – encodes a protein that contains PepSY domain; *pepS8* – encodes S8 family serine peptidase; *porV/Q* – encodes PorV/PorQ family protein; *cal* – encodes calycin-like domain-containing protein.

Our previous phylogenetic analyses demonstrated that HmuY proteins are widespread in bacteria belonging to the Bacteroidota phylum (Bielecki et al., 2018, 2020; Sieminska et al., 2021; Antonyuk et al., 2023; Śmiga and Olczak, 2024). As in *P. gingivalis*, most analyzed *Porphyromonas* species encode the *hmu* operon; however, their composition varies between species (**Figure 1**). In *Porphyromonas levii*, the locations of *hmuY* and *hmuR* genes are inverted compared to the typical *P. gingivalis hmu* operon organization. In *P. canoris*, *Porphyromonas macacae*, and *P. pogonae*, both *hmuY* and *hmuR* genes form a small operon, and the rest of the classical *hmu* operon genes (*hmuSTUV*) are encoded in a different part of the genome and form a separate gene cluster. Similar to *P. intermedia* or *B. fragilis* (Bielecki et al., 2020; Antonyuk et al., 2023), *P. canoris* and *P. pogonae* encode two HmuY homologs, and *Porphyromonas uenonis*, *Porphyromonas asaccharolytica*, and *P. cangingivalis* encode three HmuY homologs (**Figure 1**). Comparative analysis revealed that not all *Porphyromonas* species encode HmuY proteins (e.g., *P. pasteri*, *P. somerae*, *Porphyromonas bennonis*, and *P. catoniae*) (**Table 1**; **Figure 1**). Nevertheless, in *P. pasteri* and *P. somerae*, the gene homologous to *hmuR* was identified, which is part of a small gene cluster with a gene encoding a hypothetical protein. Other genes of the *hmu* operon are encoded in these species but in different regions of their genomes (**Figure 1**). As we showed previously, in some bacteria, an atypical *hmu* operon contained an additional gene located upstream of the *hmuY* gene (**Figure 1**), with *P. endodontalis* being an example (Śmiga and Olczak, 2024).

**Table 1:** A summary of main components of heme acquisition mechanisms and their activity in *Porphyromonas* species.

Species (abbreviation used in this study)	Subgroup*	Full heme biosynthesis pathway	HmuY homolog	HusA homolog	Trypsin-like activity	Gingipain homologs	Hemagglutination activity	Hemagglutinin**	Catalase activity	Pigment formation	Selected references
*P. gingivalis* (Pg)	I	–	+	+	+	+	+	+	-	+	Summanen et al., 2015; Bird et al., 2016
*P. gulae* (Pgu)	I	–	+	+	+	+	+	+	+	+	Fournier et al., 2001; Summanen et al., 2015; Bird et al., 2016
*P. loveana* (Plo)	I	–	+	+	+	+	+	+	+	+	Bird et al., 2016
*P. endodontalis* (Pe)	IIa	–	+	–	–	-	-	-	-	+	Summanen et al., 2015; Bird et al., 2016
*P. circumdentaria* (Pcd)	IIa	–	+	–	–	-	-	+	+	+	Summanen et al., 2015; Bird et al., 2016
*P. gingivicanis* (Pgc)	IIa	–	+	–	–	-	-	+	+	+	Hirasawa and Takada, 1994; Summanen et al., 2015; Bird et al., 2016
*P. crevioricanis* (Pcc)	I	–	+	–	–	-	+	-	-	+	Hirasawa and Takada, 1994; Sakamoto and Ohkuma, 2013; Summanen et al., 2015; Bird et al., 2016,
*P. levii* (Ple)	IIb	–	+	–	–	-	-	-	-	+	Summanen et al., 2015; Bird et al., 2016
*P. uenonis* (Pu)	IIa	–	+	–	–	-	NA	-	-	+	Finegold et al., 2004; Summanen et al., 2015; Bird et al., 2016
*P. asaccharolytica* (Pa)	IIa	–	+	–	–	-	-	-	-	+	Summanen et al., 2015; Bird et al., 2016
*P. cangingivalis* (Pcg)	IIb	+	+	+	–	-	-	-	+	+	Collins et al., 1994; Summanen et al., 2015; Bird et al., 2016
*P. canoris* (Pcn)	IIb	+	+	–	–	-	-	-	+	+	Love et al., 1994; Summanen et al., 2015; Bird et al., 2016
*P. macacae* (Pm)	IIb	–	+	+	+	-	-	-	+	+	Love, 1995; Summanen et al., 2015; Bird et al., 2016
*P. pasteri* (Ppa)	I	–	–	–	–	-	-	-	-	-	Sakamoto et al., 2015
*P. somerae* (Ps)	IIb	–	–	+	–	-	-	-	-	+	Summanen et al., 2005, 2015; Bird et al., 2016
*P. bennonis*	IIb	–	–	–	–	-	NA	-	+^1^	+^2^	Summanen et al., 2009, 2015; Bird et al., 2016
*P. catoniae*	I	–	–	–	+^3^	-	NA	-	-	-	Summanen et al., 2015; Bird et al., 2016
*P. bronchialis*	I^4^	NA	NA	NA	–	NA	NA	NA	-	-	Sato et al., 2015
*P. pogonae* (Ppo)	I	–	+	+	+	-	+	-	+	-	Kawamura et al., 2015; Kim et al., 2016; Sato et al., 2015
*P. katsikii*	IIb	NA	NA	NA	NA	NA	NA	NA	NA	+	Filioussis et al., 2015

**Figure 2:**
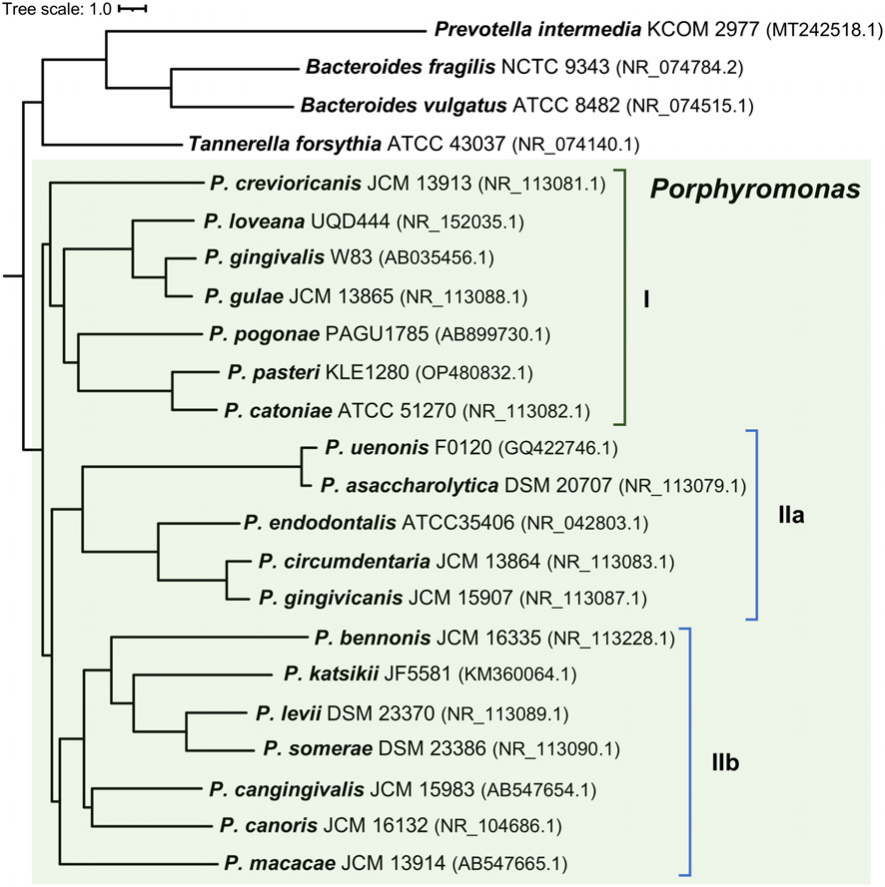
Neighbor-joining phylogenetic tree based on the *16S rRNA* gene sequences. *Porphyromonas* species can be divided into three subgroups (I, IIa, and IIb) based on their position on the phylogenetic tree. *Prevotella intermedia*, *Tannerella forsythia*, *Bacteroides fragilis*, and *Bacteroides vulgatus* were used as the out-groups. Strain names and the accession numbers of compared sequences are given next to the species names.

*grouped based on gene sequence encoding *16S rRNA* shown in **Figure 2**.

**based on *P. gingivalis* HagA homolog BLAST search.

^1^11–89% of the strains are positive for catalase activity.

^2^weak pigmentation observed after prolonged growth.

^3^11-89% of tested strains are positive for trypsin-like activity.

^4^classified based on close relation to *P. catoniae* and *P. pogonae* (Sato et al., 2015).

NA – data not available.

To examine whether the composition of the *hmu* operon correlates with the phylogenetic relationships among *Porphyromonas* species, we constructed the phylogenetic tree based on the *16S rRNA* gene sequence, allowing us to distinguish three subgroups (I, IIa, and IIb) (**Figure 2**). Their phenotypic and genomic features are summed up in **Table 1**; **Supplementary Table S4**. The characteristic features of the *Porphyromonas* species belonging to the subgroup I are hemagglutination and trypsin-like activity. The only exception is *Porphyromonas macacae*, which exhibits trypsin-like activity but belongs to subgroup IIb (**Table 1**; **Figure 2**). It is worth mentioning that homologs of *P. gingivalis* gingipains (Kgp, RgpA, and RgpB), which are highly proteolytic cysteine proteases with trypsin-like activity and considered to be the main virulence factor of this bacterium (Smalley and Olczak, 2017), were identified only in *Porphyromonas gulae* and *Porphyromonas loveana* (**Table 1**). Other features, such as the production of heme-containing pigment or catalase activity, are not unique to any subgroup. Like other Bacteroidota members, *Porphyromonas* species cannot synthesize heme *de novo*, except for *Porphyromonas cangingivalis* and *Porphyromonas canoris* (O’Flynn et al., 2015; Love et al., 1994) (**Table 1**).

### 3.2 HmuY proteins differ among members of the *Porphyromonas* genus

**Figure 3:**
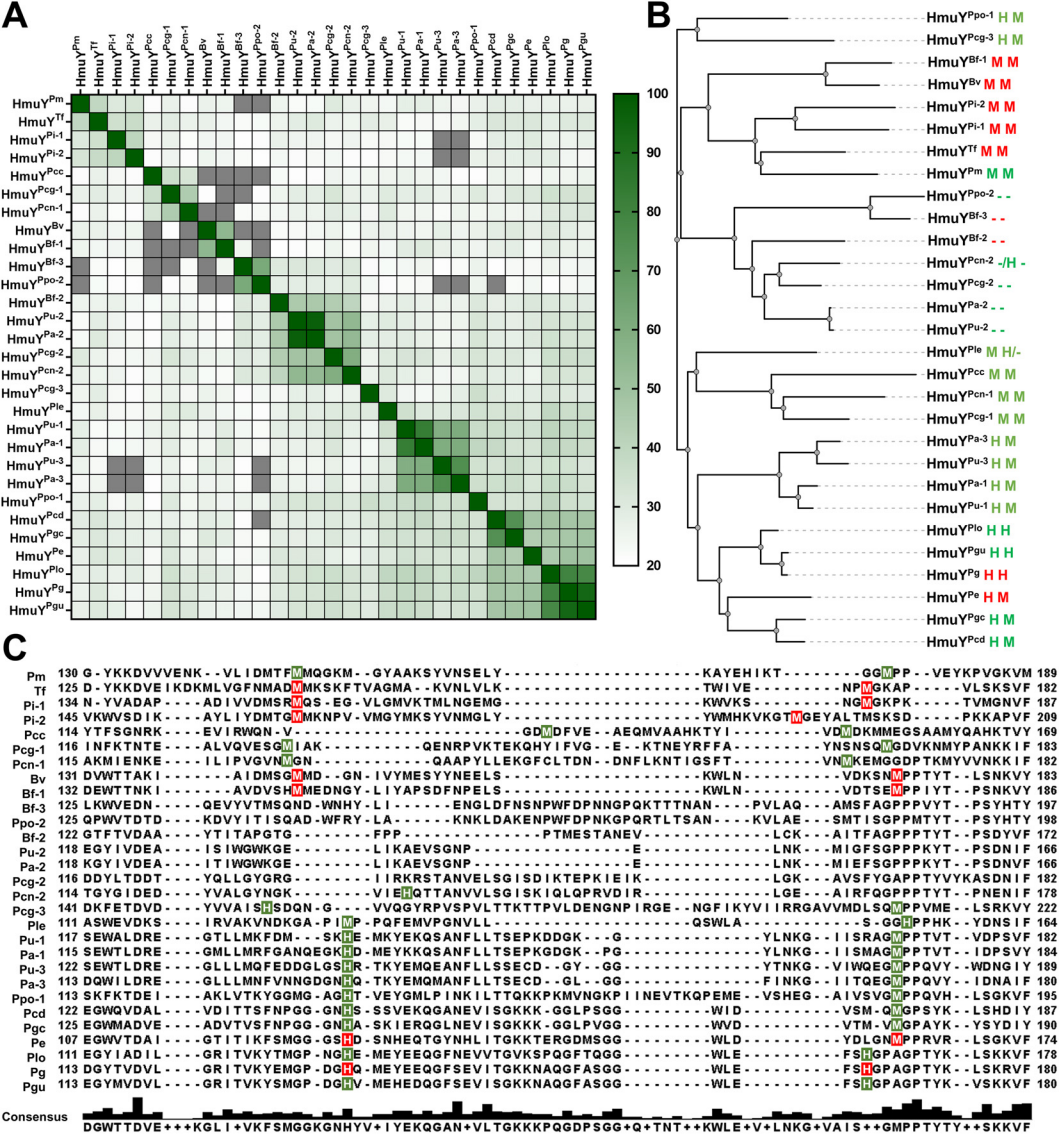
Comparative analysis of HmuY family proteins of *Porphyromonas* species and other representative members of the Bacteroidota phylum. **(A)** A heat map was constructed based on the amino acid sequence of HmuY proteins. The color gradient from white to dark green shows the percentage of identity from lowest (20%) to highest (100%). Values below 20% are shown in grey. **(B)** The phylogenetic tree was constructed based on the amino acid sequence of HmuY proteins. Amino acids involved in heme-iron coordination confirmed experimentally are shown in red, and their counterparts predicted based on the amino acid sequences and three-dimensional modeled protein structures are shown in green. The lack of amino acids coordinating heme-iron is shown as a dash (-). **(C)** Amino acid sequence alignment of the selected regions of HmuY family proteins. Amino acids involved in heme-iron coordination are shadowed in red (experimentally confirmed) or green (predicted). The consensus amino acid sequence is shown below the examined sequences. Species names with abbreviations, given along with HmuY names, are listed in **Table 1** and **Supplementary Table S4**.

**Figure 4:**
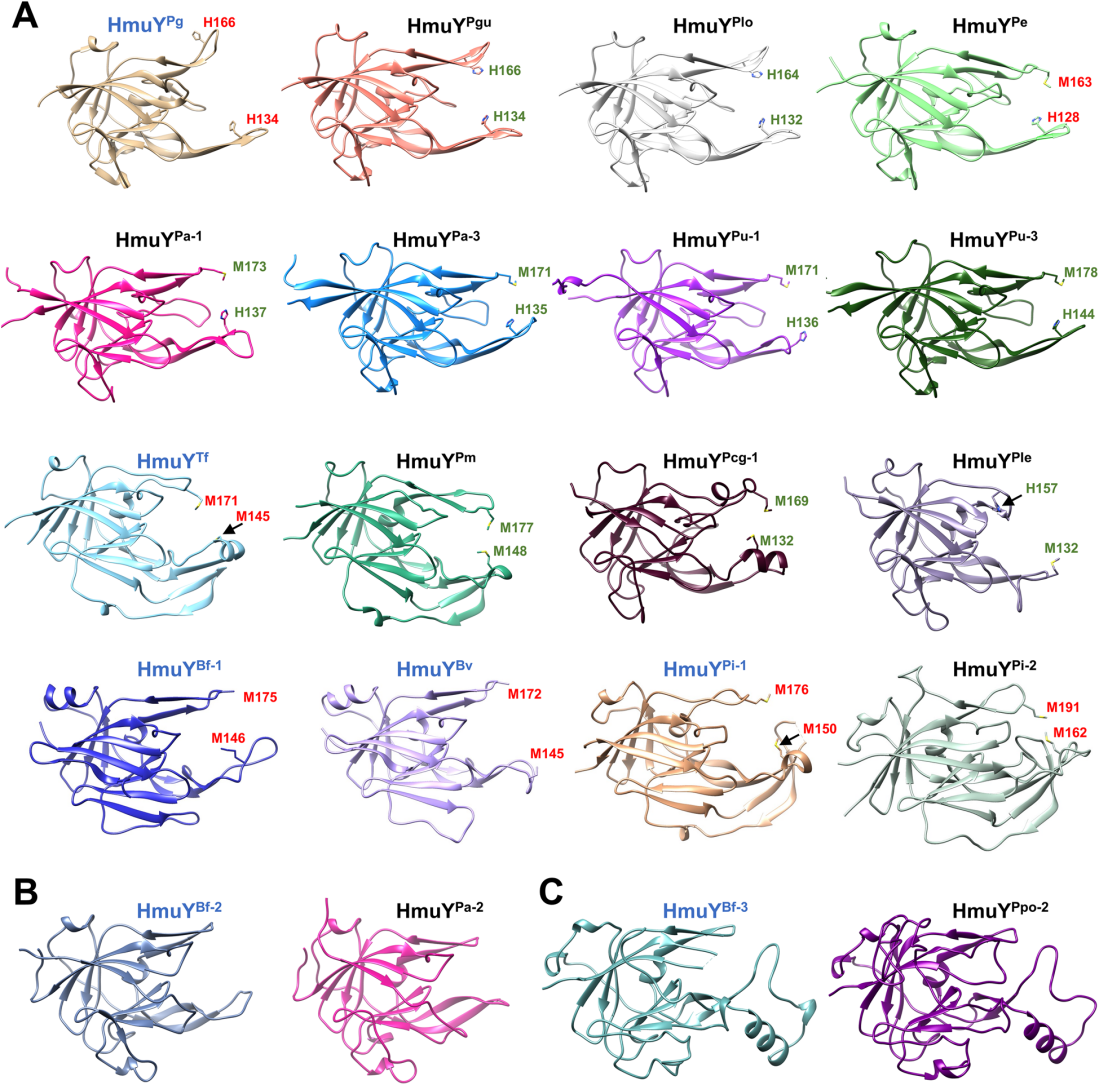
Comparison of overall three-dimensional structures of selected HmuY apo-proteins. The structure of HmuY proteins consists mostly of β strands forming the conserved core region. The most diverse part of the HmuY proteins is their ligand-binding pocket. Names of protein structures determined by crystallography are shown in blue, and the names of modeled structures of other HmuY proteins are shown in black. **(A)** HmuY homologs with predicted (green) or experimentally identified amino acids (red) involved in heme-iron coordination. **(B)** HmuY homologs with atypical heme binding, exhibiting a higher preference for binding PPIX over heme without heme-iron coordination (HmuY^Bf-2^ and HmuY^Pa-2^), and **(C)** HmuY homologs which bind neither heme nor PPIX (HmuY^Bf-3^ and HmuY^Ppo-2^). PDB IDs: HmuY^Pg^, 6EWM; HmuY^Tf^, 6EU8; HmuY^Bf-1^, 4GBS; HmuY^Bv^, 3U22; HmuY^Pi-1^, 6R2H; HmuY^Bf-2^, 8B6A; HmuY^Bf-3^, 8B61. Three-dimensional structures of other proteins were predicted using AlphaFold (https://alphafold.com) (Jumper et al., 2021; Varadi et al., 2022). Protein structures were visualized with UCSF Chimera (https://www.cgl.ucsf.edu/chimera/) (Pettersen et al., 2004).

Based on the amino acid sequence, HmuY homologs show a low identity (**Figure 3A**; **Supplementary Figure S2**). A phylogenetic analysis revealed a few main groups that, besides higher amino acid sequence identity within a group, demonstrate the same heme-iron coordinating amino acid pair (**Figures 3B, C**; **Supplementary Figure S2**). Only proteins closely related to the HmuY^Pg^, namely HmuY^Pgu^ from *P. gulae* and HmuY^Plo^ from *P. loveana*, might coordinate heme-iron using two histidines (**Figures 3B, C**, **4A**). Most of the other HmuY homologs identified in *Porphyromonas* species may use two methionines or a pair of methionine-histidine (**Figures 3B, C**, **4A**).

*Porphyromonas* species encoding more than one HmuY homolog possess proteins with high identity to *B. fragilis* HmuY^Bf-2^ (HmuY^Pcn-2^, HmuY^Pcg-2^, HmuY^Pa-2^, and HmuY^Pu-2^) or HmuY^Bf-3^ (HmuY^Ppo-2^) (**Figures 1**, **3**), whose function may be different compared to classical heme-binding HmuY family members (Olczak et al., 2024). They possess neither histidine nor methionine in the corresponding positions involved in heme-iron coordination in other HmuY proteins (**Figures 3B, C**, **4B, C**).

Similar to *B. fragilis* HmuY^Bf-2^, *P. gingivalis* HusA^Pg^ protein binds heme and PPIX (Antonyuk et al., 2023; Gao et al., 2010, 2018; Śmiga et al., 2023a). HusA^Pg^ homologs have only been found in a few *Porphyromonas* species (**Table 1**; **Supplementary Figure S3**). Interestingly, their presence does not correlate with the organization of *hmu* operons or *hmu* gene clusters or the number of genes encoding HmuY proteins (**Figure 1**; **Supplementary Figure S3A**; **Table 1**). However, the *husA* gene is absent in *Porphyromonas* species that encode a homolog of HmuY^Bf-2^. The only exception is *P. cangingivalis*, which possesses three *hmuY* genes and the *husA* gene (**Figure 1**; **Supplementary Figure S3A**; **Table 1**).

### 3.3 HmuY proteins differ in heme coordination modes

To investigate the correlation between the type of ligand-binding amino acids and the heme- and PPIX-binding ability, representative proteins were selected: HmuY^Pg^ with a histidine pair (His-His), HmuY^Pe^ with a histidine-methionine pair (His-Met), HmuY^Tf^ with a methionine pair (Met-Met), and HmuY^Bf-2^ with no residues involved in heme-iron coordination. Additionally, as a control, we selected the HusA^Pg^ protein, which binds heme and PPIX (Gao et al., 2018).

The spectroscopic methods are usually employed to analyze the binding mode of heme or PPIX, where the Soret (~400 nm) and Q bands (~500–600 nm) maxima of the UV-visible spectra are characteristic of the respective complexes formed. Soret band arises from π-π* S0→S2 transition (singlet ground state to second excited single state), whereas Q bands arise from π-π* S0→S1 transition (singlet ground state to first excited single state). Four clear transitions in the Q band region are identified in metal-free porphyrins and two transitions in metalloporphyrins (Giovanetti, 2012), allowing differentiation between heme and PPIX binding.

**Figure 5:**
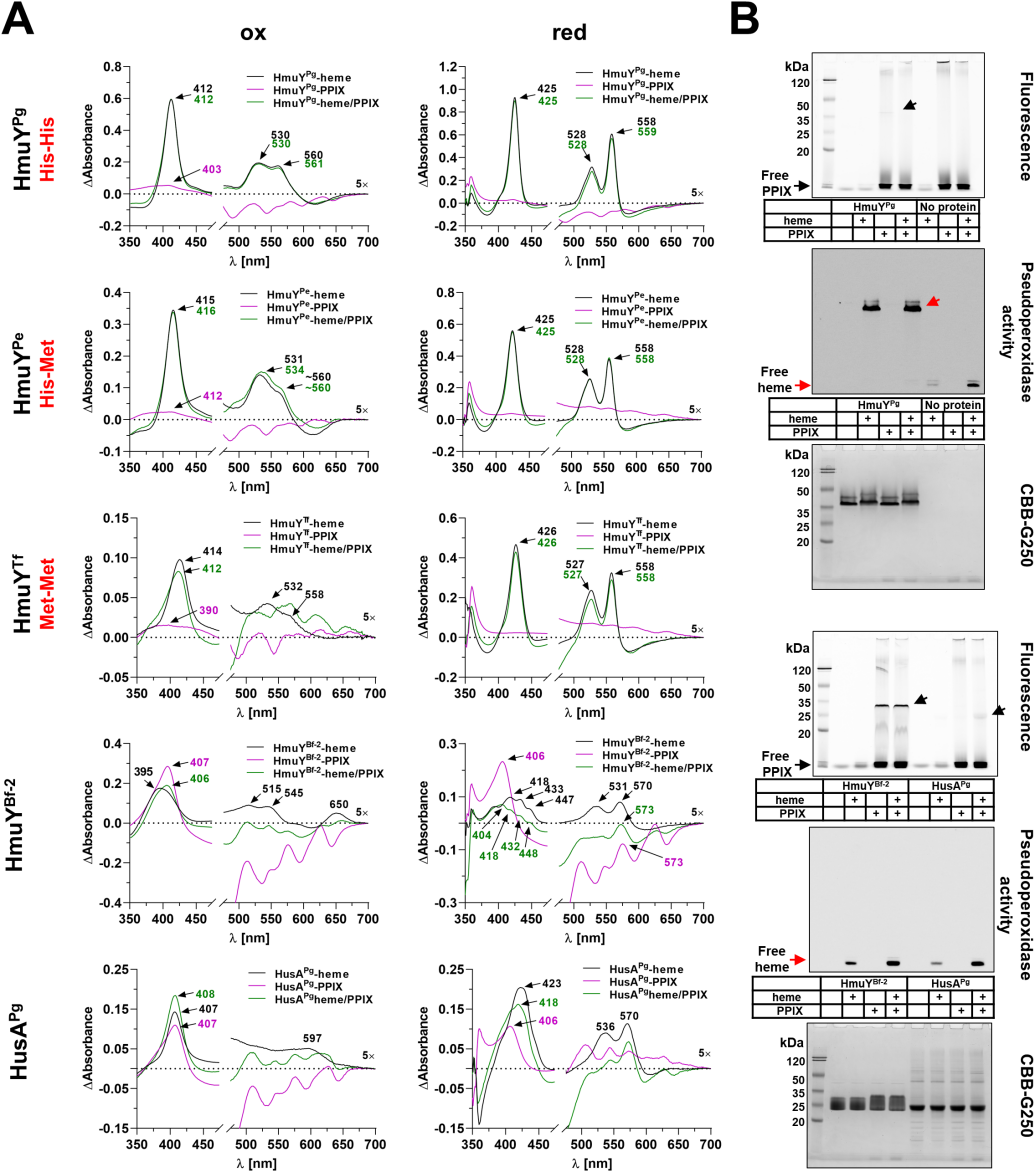
Heme- and PPIX-binding capacity. *Porphyromonas gingivalis* (HmuY^Pg^), *Porphyromonas endodontalis* (HmuY^Pe^), and *Tannerella forsythia* (HmuY^Tf^), or *Bacteroides fragilis* (HmuY^Bf-2^) and *P. gingivalis* HusA proteins were selected to demonstrate differences in heme alone (black lines), PPIX alone (purple lines), or heme in the presence of PPIX (green lines) binding, resulting from the presence of two histidines (His-His), a histidine-methionine pair (His-Met), two methionines (Met-Met), or the absence of amino acids coordinating heme-iron. The binding was examined using difference absorbance spectroscopy (ΔAbsorbance) **(A)** or PAGE (semi-denaturing conditions) and subsequent fluorescence or pseudoperoxidase activity visualization, and final staining with CBB-G250 **(B)**. Absorbance spectra were recorded using 5 µM proteins and 5 µM porphyrins under oxidizing (ox) or reducing (red) conditions, the latter conditions formed by 10 mM sodium dithionite. Arrows indicate free heme and heme bound to proteins (red) or free PPIX and PPIX bound to proteins (black).

In this study, we demonstrated that PPIX alone is not efficiently bound by HmuY^Pg^, HmuY^Pe^, and HmuY^Tf^ (**Figure 5A**; **Supplementary Figure S4**), and as shown previously (Bielecki et al., 2018; Śmiga and Olczak, 2024), heme binding to HmuY^Pg^ was the strongest under oxidizing conditions, while HmuY^Tf^ showed the weakest binding under these conditions. Under reducing conditions, however, HmuY^Pg^, HmuY^Pe^, and HmuY^Tf^ bound heme efficiently (**Figure 5A**; **Supplementary Figure S4**). The heme-binding was stronger even in the presence of PPIX, thus confirming the specificity of heme over PPIX binding (**Figure 5A**; **Supplementary Figure S4**). In contrast, the opposite effect was observed for HmuY^Bf-2^, which, similar to HusA^Pg^, binds both heme and PPIX. However, the spectra obtained under both oxidizing and reducing conditions for heme, PPIX, and their mixture showed that both proteins, especially HmuY^Bf-2^, prefer the binding of the metal-free PPIX ring (**Figure 5A**; **Supplementary Figure S4**). Experiments using mesoporphyrin (MPIX) or mesoheme (FeMPIX) confirmed results obtained for heme and PPIX binding (**Supplementary Figures S5**, **S6**). Data gained by UV-visible absorbance spectroscopy were confirmed for HmuY^Pg^, HmuY^Bf-2^, and HusA^Pg^ by PAGE carried out under semi-denaturing conditions and subsequent fluorescence (PPIX visualization) or pseudoperoxidase activity (heme visualization) detection (**Figure 5B**). Heme binding by HmuY^Pg^ and PPIX binding by HmuY^Bf-2^ were detected after electrophoretic separation of proteins and porphyrins. The specific ligand binding also affected the protein migration under semi-denaturing conditions, as evidenced by the slower migration of HmuY^Pg^-heme and HmuY^Bf-2^-PPIX complexes in comparison to the apo-protein forms (**Figure 5B**). In contrast, no evidence of HusA-heme or HusA-PPIX complex formation was observed using this method, neither through fluorescence/chemiluminescence detection nor changes in protein migration (**Figure 5B**).

### 3.4 Histidines may be an evolutionarily gained advantage in heme-iron coordination of HmuY and HmuR proteins

**Figure 6:**
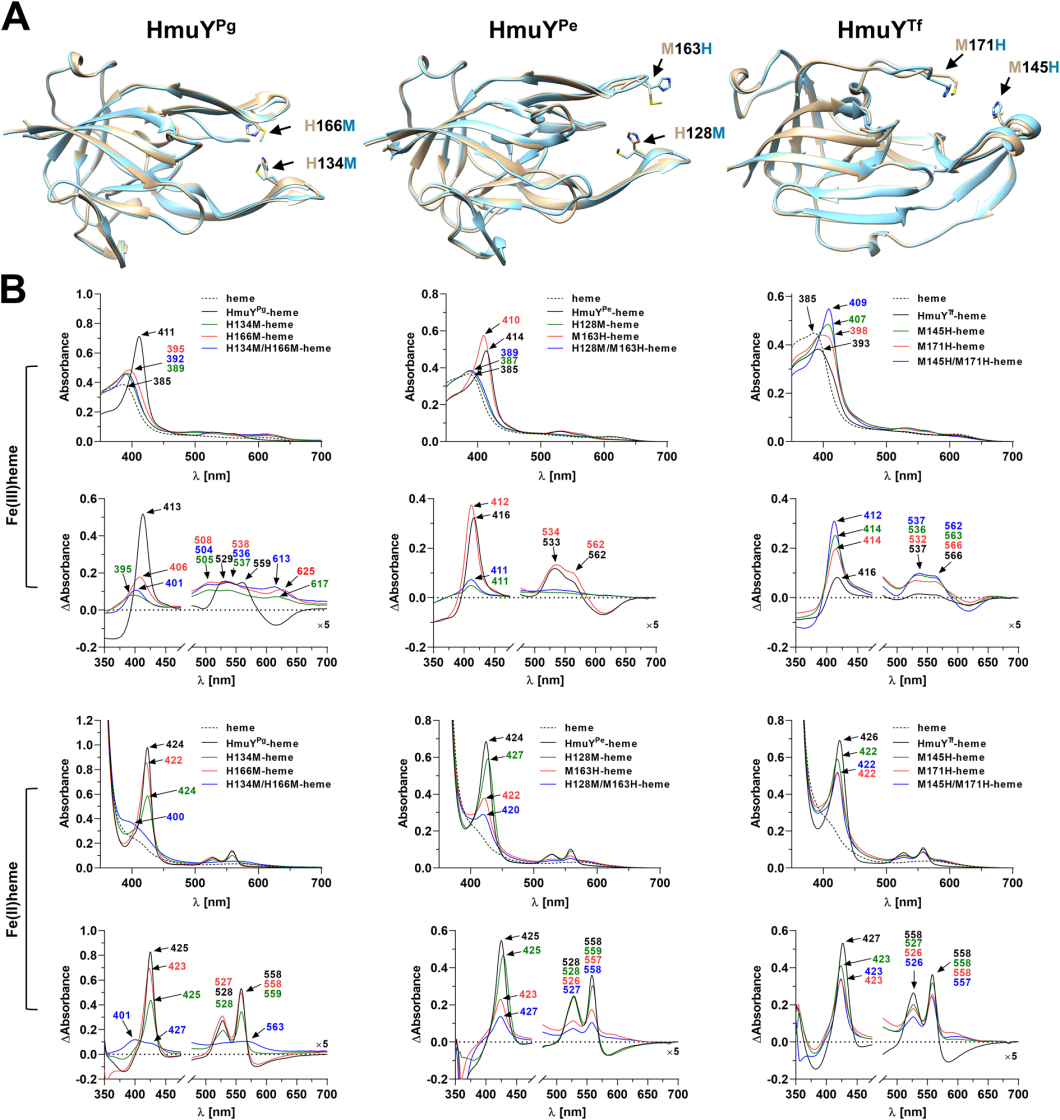
Heme binding to site-directed mutagenesis variants of HmuY^Pg^, HmuY^Pe^, and HmuY^Tf^ proteins. **(A)** Comparison of the superimposed three-dimensional structures of unmodified proteins (beige) and site-directed mutagenesis variants (blue). Amino acids involved in heme binding are marked with arrows in three-dimensional structures of the *Porphyromonas gingivalis* HmuY^Pg^-heme complex (PDB ID: 3H8T), *Porphyromonas endodontalis* apo-HmuY^Pe^ (modeled protein structure), and *Tannerella forsythia* apo-HmuY^Tf^ (PDB ID: 6EU8). **(B)** Heme binding to site-directed mutagenesis variants of HmuY^Pg^, HmuY^Pe^, and HmuY^Tf^ was examined by UV-visible spectroscopy and difference absorption spectroscopy (ΔAbsorbance). Spectra were recorded under oxidizing (Fe(III)heme) and reducing (Fe(II)heme) conditions, the latter conditions formed by 10 mM sodium dithionite. Spectra of heme were recorded to demonstrate differences between heme alone and protein-heme complexes. The structure of apo-HmuY^Pe^ was modeled with AlphaFold (https://alphafold.com) (Jumper et al., 2021; Varadi et al., 2022). The site-directed mutagenesis protein variants were modeled with Phyre2 (Kelley et al., 2015) and subsequently refined using ModRefiner (Xu and Zhang, 2011). Protein structures were visualized with UCSF Chimera (https://www.cgl.ucsf.edu/chimera/) (Pettersen et al., 2004).

Although histidine or methionine residues are used to bind heme, it seems that individual HmuY protein prefers a specific combination of amino acids coordinating heme-iron efficiently. Our preliminary studies demonstrated that the modified HmuY^Pg^ protein with two methionines instead of two histidines showed significantly lower heme-binding capacity than the unmodified protein, especially under oxidizing conditions (Kosno et al., 2022). To find whether the substitution of methionine with histidine would affect heme binding to other proteins, we examined HmuY^Pe^ and HmuY^Tf^ site-directed mutagenesis variants and compared them with HmuY^Pg^ variants (**Figure 6A**). In HmuY^Pe^, the substitution of single methionine by histidine (M163H), resulting in two histidines coordinating heme-iron, improved heme binding under oxidizing conditions (**Figure 6B**), while under reducing conditions, it resulted in a decreased ability to bind heme. A similar effect was observed in the case of the H128M/M163H variant. Only the H128M variant bound heme under reducing conditions with similar efficiency as compared to the unmodified protein, probably due to the presence of two methionines coordinating heme-iron. Substitution of two methionines by histidines in HmuY^Tf^ (M145H/M171H) increased heme binding under oxidizing conditions (**Figure 6B**). Single substitution of methionines by histidines also improved heme binding, but to a lesser degree. Under reducing conditions, however, all protein variants bound heme with a lower ability compared to the unmodified protein, and this effect was mostly seen in the case of the M145H/M171H protein variant.

Also, HmuR proteins encoded next to HmuY proteins may use different amino acid combinations for heme-iron coordination (Antonyuk et al., 2023). The extended analysis using HmuR homologs of *Porphyromonas* species showed that most of them encode TDRs with two histidines predicted to bind heme, even in species where the HmuY protein uses a histidine-methionine pair (**Supplementary Figures S7**, **S8**). This additionally indicates the superiority of histidine over methionine in the heme uptake systems of these bacteria.

### 3.5 Tyrosine residues of HmuY and Hus proteins are important in the binding of the protoporphyrin IX ring

**Figure 7:**
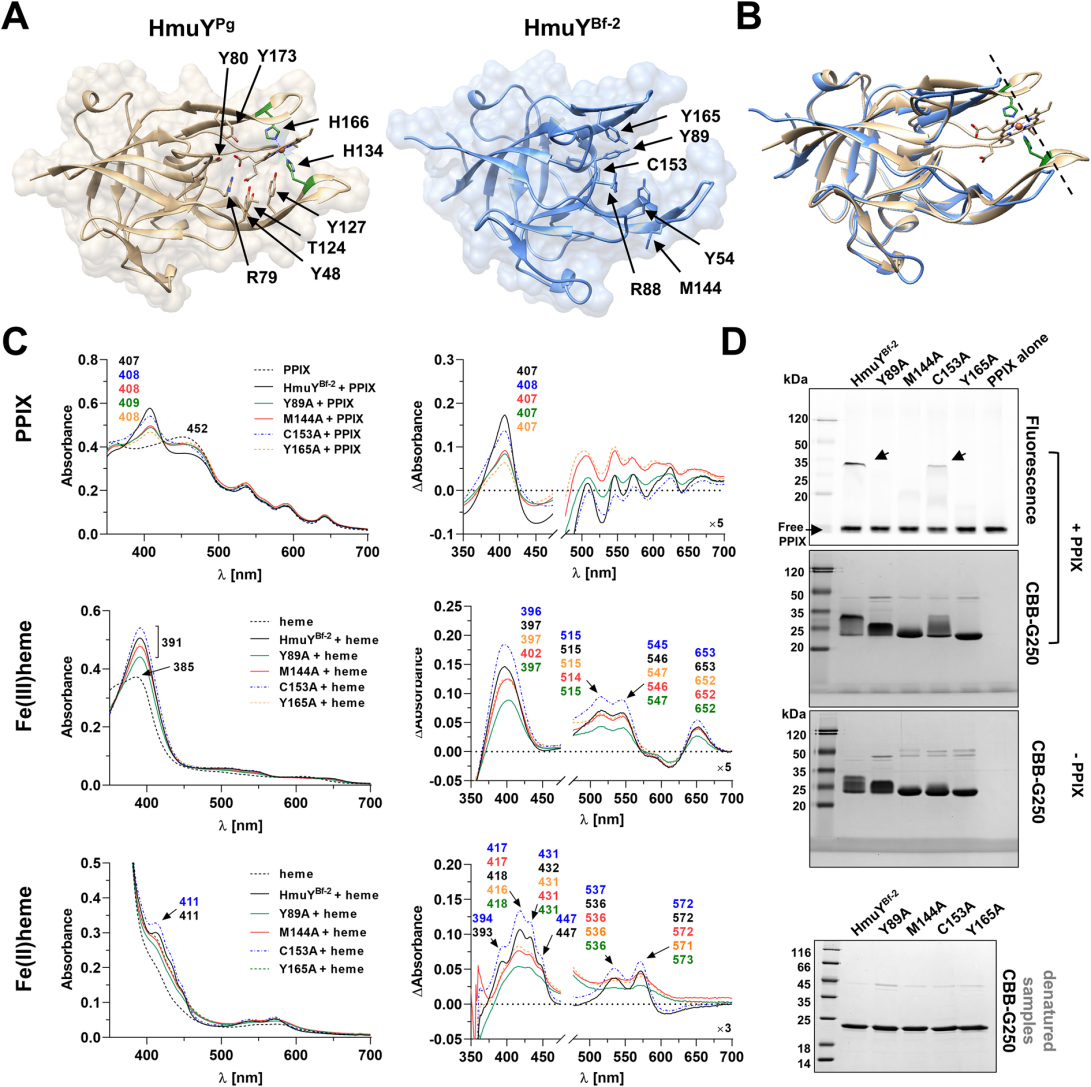
Comparison of amino acids involved in binding of the PPIX ring in *Porphyromonas gingivalis* HmuY^Pg^ and *Bacteroides fragilis* HmuY^Bf-2^. **(A)** Amino acids involved in PPIX binding are marked with arrows in three-dimensional structures of the HmuY^Pg^-heme complex (PDB ID: 3H8T) and apo-HmuY^Bf-2^ (PDB ID: 8B6A). **(B)** The superimposition of both protein structures demonstrates differences in the heme/PPIX-binding pockets, with shorter loops of the ligand-binding pocket in HmuY^Bf-2^ (shown by the dashed line). The binding of PPIX and heme to site-directed mutagenesis variants of HmuY^Bf-2^ was examined by UV-visible spectroscopy, difference absorption spectroscopy (ΔAbsorbance) **(C)**, and the binding of PPIX also by PAGE (semi-denaturing conditions) and fluorescence visualization with subsequent Coomassie Brilliant Blue G-250 staining (CBB-G250) **(D)**. SDS-PAGE (denatured samples) and CBB-G250 staining were carried out as a control. The binding of heme was examined under oxidizing (Fe(III)PPIX) and reducing (Fe(II)PPIX) conditions, the latter conditions formed by 10 mM sodium dithionite. Spectra of PPIX or heme demonstrate differences between PPIX or heme alone and protein-PPIX or protein-heme complexes.

Although amino acid sequences of HmuY proteins display relatively low identity, particularly between distant species (**Figure 3**; **Supplementary Figure S2**), the overall three-dimensional protein structures exhibit significant similarity (**Figure 4**; **Supplementary Table S1**). As we showed previously (Śmiga et al., 2023b), most conserved fragments are found in the core regions of HmuY proteins (**Supplementary Figure S2**). In contrast, a lower amino acid sequence identity is observed in the fragments that form heme-binding pockets (**Figure 3C**, **Supplementary Figure S2**). This results not only in different heme-iron coordinating amino acids, as mentioned above, but also in different structures of heme-binding pockets (**Figures 4**, **7A**), including shorter loops forming the binding pocket in HmuY^Bf-2^ and its closest homologs (**Figures 4**, **7B**). Analysis of the experimentally solved structure of the HmuY^Pg^-heme complex revealed amino acids engaged in the binding of the PPIX ring (selected conserved amino acids are indicated in **Figure 7A**; **Supplementary Figure S2**), with tyrosine residues being dominant (Wojtowicz et al., 2009b). Many of them, especially tyrosines, can be found in HmuY proteins capable of heme or PPIX binding, including HmuY^Bf-2^ (**Figure 7A**; **Supplementary Figure S2**). HmuY^Bf-2^ binds heme without heme-iron coordination, most likely using interactions between amino acid residues from the ligand-binding pocket and the PPIX ring (Antonyuk et al., 2023). Therefore, this protein is an excellent research model for analyzing the influence of tyrosines located inside the ligand-binding pocket on the interaction with PPIX. UV-visible absorbance spectroscopy examination of site-directed mutagenesis variants of HmuY^Bf-2^ with selected amino acids substituted with alanine (Y89A, M144A, C153A, and Y165A) confirmed the decreased capacity of heme and PPIX binding except for cysteine (C153), which is not located in the ligand-binding pocket and therefore was used as a control (**Figure 7**). Both analyzed tyrosines (Y89 and Y165) are highly conserved in HmuY proteins (**Supplementary Figure S2**) and may play a critical role in heme/PPIX binding. Their substitution with alanine decreased heme/PPIX binding (**Figure 7C**). Additionally, we confirmed these results with PAGE carried out under semi-denaturing conditions and subsequent fluorescence PPIX visualization. Only the unmodified HmuY^Bf-2^ and the C153A variant showed PPIX binding with simultaneous retardation of the fraction of the protein migration in the gel. Similarly, the engagement of tyrosine in heme/PPIX binding was shown for *P. gingivalis* HusA^Pg^, which uses only one tyrosine and other different amino acids (Gao et al., 2018). Moreover, a comparison with the sequences of HusA homologs from other *Porphyromonas* species showed that tyrosine involved in heme/PPIX binding is highly conserved (**Supplementary Figure S3**), similar to tyrosine residues present in the binding pocket of HmuY proteins.

## 4 Discussion

Bacterial virulence is related to environmental conditions that can affect the selection of specific features. Adaptation to the occupied niche or host may have driven changes that increase the efficiency of heme acquisition by different *Porphyromonas* species, allowing better adaptation and higher pathogenicity. Hmu systems and especially HmuY proteins may have evolved to specialize in heme uptake in various host niches and under different heme availabilities to meet the heme requirements of specific *Porphyromonas* species. It can be hypothesized that genes encoding proteins forming the Hmu system, specifically focusing on HmuY and HmuR, may exhibit high susceptibility to mutations and the dynamic evolutionary events of gene duplication and/or transfer across species. In multispecies microbial consortia, particular genes may have been recombined between species, lost, acquired, or duplicated. Based on data collected in this study, we assume that these processes may have occurred in *Porphyromonas* species independently multiple times. Adaptation for heme acquisition and interspecific competition has most likely led not only to genes’ duplication or re-location but also to the development of the unique structure and function of HmuY^Pg^, as compared to homologous proteins produced by other members of the *Porphyromonas* genus, and in the wider context, by members of the Bacteroidota phylum. Such an advantage may result in better adaptation to the changing conditions of niches *P. gingivalis* occupies and its higher virulence potential over cohabitating bacteria. Moreover, high diversity among HmuY homologs may result in their variable function (Olczak et al., 2024). Additionally, differences in the location of *hmuY* and *hmuR* genes in the respective genomes may influence the pathogenic potential of *Porphyromonas* species. The organization of *hmu* operons and *hmu*-like gene clusters differs not only between the members of the Bacteroidota phylum but also between *Porphyromonas* species to an even higher extent, which might be responsible for changes in their adaptation to the host niches.

Differences between bacteria may also exist in different expressions of particular genes. Expression of the *hmu* operon genes in *P. gingivalis* and other bacteria is higher in heme and iron-depleted conditions, is dependent on the growth phase, and is regulated by several transcription factors (Smalley and Olczak, 2017; Olczak et al., 2024). Moreover, the expression of the *hmu* operon genes, best characterized in *P. gingivalis*, differs (Lewis et al., 2006; Olczak et al., 2008). The complexity of the production of the *hmu* operon transcript can be explained, at least in part, by the formation of secondary mRNA structures located between *hmuY* and *hmuR* and between *hmuR* and *hmuS* sequences (Lewis et al., 2006). In other *Porphyromonas* species, where *hmu* operon genes are encoded in different loci of their genomes and bacteria produce more than one HmuY homolog (**Figure 1**), the regulation process may be different and even more complex. Therefore, the production of Hmu system proteins may also depend on the location of the *hmuY* gene in the operon and the composition of the operon itself. The complexity of gene expression regulation may be a factor that improves the potential of bacteria to adapt to changing environmental conditions.

Another important difference is the ability of heme binding. The strength of heme binding by HmuY proteins depends on the redox state of the external environment (Olczak et al., 2024). HmuY^Pg^, coordinating heme-iron with two histidines, binds heme efficiently under oxidizing and reducing conditions with dissociation constants *K_d_* < 10^–9^ and *K_d_* ~10^-8^, respectively (Bielecki et al., 2018). When two methionines or a histidine-methionine pair are involved in this process in HmuY homologs, they bind heme preferentially under reducing conditions with *K_d_* ~10^-9^ (eg. HmuY^Tf^, HmuY^Pi-1^, HmuY^Pi-2^, HmuY^Bf-1^, HmuY^Bv^) (Bielecki et al., 2018, 2020, Antonyuk et al., 2023; Sieminska et al., 2021) and *K_d_* ~10^–8^ for HmuY^Pe^ (Śmiga and Olczak, 2024). However, under oxidizing conditions, HmuY^Pe^ binds heme with higher efficiency (*K_d_* ~10^-7^) as compared to HmuY^Tf^ (*K_d_* ~10^-6^) (Bielecki et al., 2018; Śmiga and Olczak, 2024). This can be explained, at least in part, by the theory of hard and soft acids and bases (Pearson, 1963), according to which the better ability of heme binding to proteins utilizing methionine instead of histidine to coordinate heme-iron under reducing conditions is observed (Pearson, 1963; Ayers et al., 2006). Heme released from hemoproteins can be easily oxidized; therefore, bacteria using the HmuY protein with histidines involved in heme binding have an advantage even under oxidizing conditions. Moreover, HmuY^Pg^ but not other HmuY proteins (including HmuY^Pe^, HmuY^Tf^, HmuY^Pi-1^, HmuY^Pi-2^, HmuY^Bf-1^, and HmuY^Bv^) directly sequester heme from methemoglobin, which is most likely possible thanks to two histidines in the binding pocket.

The experimental analyses using the substitution of amino acids engaged in heme-iron coordination indicate that the replacement of methionine with histidine increases the ability of heme binding by HmuY proteins under oxidizing conditions. On the other hand, the substitution of histidines with methionines does not always increase heme binding ability under reducing conditions, and the best example is the HmuY^Pg^ H134M/H166M variant. This effect could result from differences in the structures of particular amino acids, resulting in small changes in their spatial location, which may cause local subtle structural changes in the entrance to the heme-binding pocket (**Figure 6A**). Based on our findings, one may assume that HmuY proteins differently tolerate the substitution of particular amino acids involved in heme-iron coordination.

When heme is not available, bacteria from the Bacteroidota phylum can grow in the presence of PPIX and iron (Gao et al., 2018; Antonyuk et al., 2023; Śmiga et al., 2024), suggesting they can synthesize heme from these components. In the host body, excess PPIX may be released from the mitochondria to the cytoplasm and then from the cell into the extracellular space. Moreover, PPIX excess can accumulate in red blood cells and can also be present in small amounts in serum or serum exudates (Kiening and Lange, 2022; Sachar et al., 2016). Therefore, during the adaptation of the binding pocket of a group of HmuY proteins, they developed an ability to bind PPIX, which broadens the heme uptake strategy. Moreover, this process may have also occurred in the case of other hemophore-like proteins, HusA being an example.

Also, the environment of the binding pocket is important for efficient heme/PPIX binding to HmuY proteins. As shown for HmuY^Pg^, its Tyr54, Tyr165, Tyr89, Arg88, and Thr133 residues are engaged in the binding of the PPIX ring. They are especially crucial for HmuY proteins that prefer to bind PPIX over heme, such as HmuY^Bf-2^. Taking into account our (Antonyuk et al., 2023 and this study) and others’ observations (Gao et al., 2018), the number of tyrosine residues engaged in porphyrin binding may influence the strength of their interaction. It could explain the higher efficiency of PPIX binding to HmuY^Bf-2^ (*K_d_* ~10^-8^) (Antonyuk et al., 2023) than to HusA^Pg^ (*K_d_* ~10^-6^), which uses in this process two and one tyrosine residue, respectively. The strength and specificity of the porphyrin binding of HmuY proteins may also depend on the shape of the binding pocket. For example, in HmuY^Bf-2^ and its closest homologs from *Porphyromonas* species (e.g., HmuY^Pa-2^) loops forming the entrance to the heme-binding pocket are slightly shorter than in HmuY^Pg^ (**Figures 4B**, **7B**), potentially facilitating the interaction of the PPIX ring with amino acids located at the back of the ligand-binding pocket. In addition, HmuY^Bf-3^ and its closest homolog from *P. pogonae* (HmuY^Ppo-2^) possess an unusual loop with an alpha-helix (**Figure 4C**), which might block the entrance of heme or PPIX to the ligand-binding pocket. However, the inability of heme or PPIX binding to HmuY^Bf-3^ may also be explained by different amino acids inside the ligand-binding pocket where only one conserved tyrosine (Y88) is present. Nevertheless, the composition of amino acid content and the shape of the ligand-binding pocket of HmuY proteins can determine the specificity of the bound ligands, such as heme in the case of classical HmuY^Pg^ and metal-free porphyrins, like PPIX in the case of HmuY^Bf-2^.

As stated above, *P. gingivalis* has developed sophisticated strategies allowing more efficient utilization of various heme sources in hostile environments and efficient competition with cohabitating bacteria. This also includes a unique accessory gingipain-Hmu system-based mechanism that facilitates heme acquisition from erythrocytes and hemoproteins (Smalley et al., 2011; Śmiga et al., 2023a). Interestingly, homologs of these cysteine proteases have been identified only in two other *Porphyromonas* species, *P. gulae* and *P. loveana*, which are canine and marsupial pathogens, respectively (Morales-Olavarria et al., 2023). Therefore, in the human oral microbiome, gingipains are characteristic only of *P. gingivalis*. This may explain why *P. gingivalis* is predisposed to play a key pathogen role in dysbiosis within the human microbiome regardless of redox conditions, a greater ability to onset and progress periodontitis, and participation in comorbidities.

Increased *P. gingivalis* resistance to antibiotics (Conrads et al., 2021; Abe et al., 2022; Ng et al., 2024; Rams et al., 2023) forces the search for alternative periodontitis treatment methods. *P. gingivalis* susceptibility to metronidazole depends on heme availability since some strains displayed differential expression of iron and heme uptake systems (Li et al., 2018; Seers et al., 2020). Therefore, elaborating diagnostic and treatment methods using components of heme uptake as a target appears promising. For example, non-iron metalloporphyrins, exploiting heme uptake systems due to the Trojan horse strategy, exhibit potent antibacterial activity (Stojiljkovic et al., 1999; Wojaczynski et al., 2011; Yukitake et al., 2011; Olczak et al., 2012; Wojtowicz et al., 2013). Due to differences in heme/PPIX-binding ability, they may be used to specifically target particular *Porphyromonas* species.

## Data Availability

The original contributions presented in the study are included in the article/**Supplementary Material**. Further inquiries can be directed to the corresponding author/s.
