# Supplementary material for: HmuY proteins of the Porphyromonas genus show diversity in heme-binding properties
Source: Front Cell Infect Microbiol. 2025 May 13;15:1560779. doi: 10.3389/fcimb.2025.1560779 (PMC12106395; doi:10.3389/fcimb.2025.1560779)
Supplement: Supplementary file 1 [file DataSheet1.pdf]

Supplemental data for:

HmuY proteins of the *Porphyromonas* genus show diversity in heme-binding properties

Michał Śmiga*, Teresa Olczak

Laboratory of Medical Biology, Faculty of Biotechnology, University of Wrocław, 50-383 Wrocław, 14A F. Joliot-Curie St., Poland

*Correspondence: Michał Śmiga; E-mail: michal.smiga@uwr.edu.pl

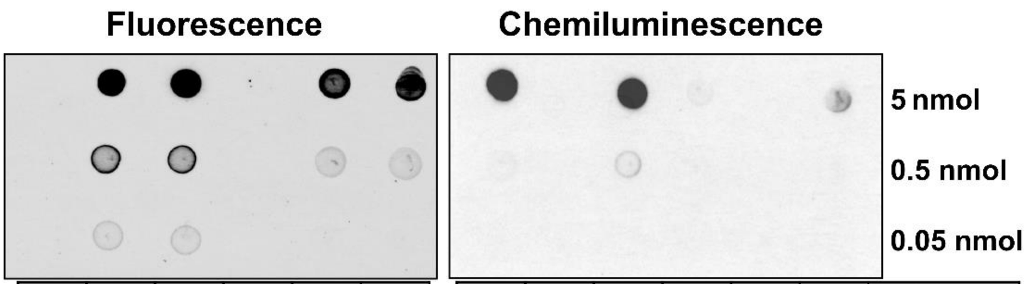

+		+				+		+				Fe(III)PPIX
	+	+					+	+				PPIX
			+		+				+		+	Fe(III)MPIX
				+	+					+	+	MPIX

Fig. S1. Confirmation of the suitability of fluorescence or chemiluminescence detection of PPIX/MPIX or heme (FePPIX)/mesoheme (FeMPIX), respectively. 5 µl of the respective porphyrin solution in PBS was applied onto the nitrocellulose membrane and fluorescence (Epi-far red) or chemiluminescence was detected using the ChemiDoc Imaging System (Bio-Rad Laboratories).

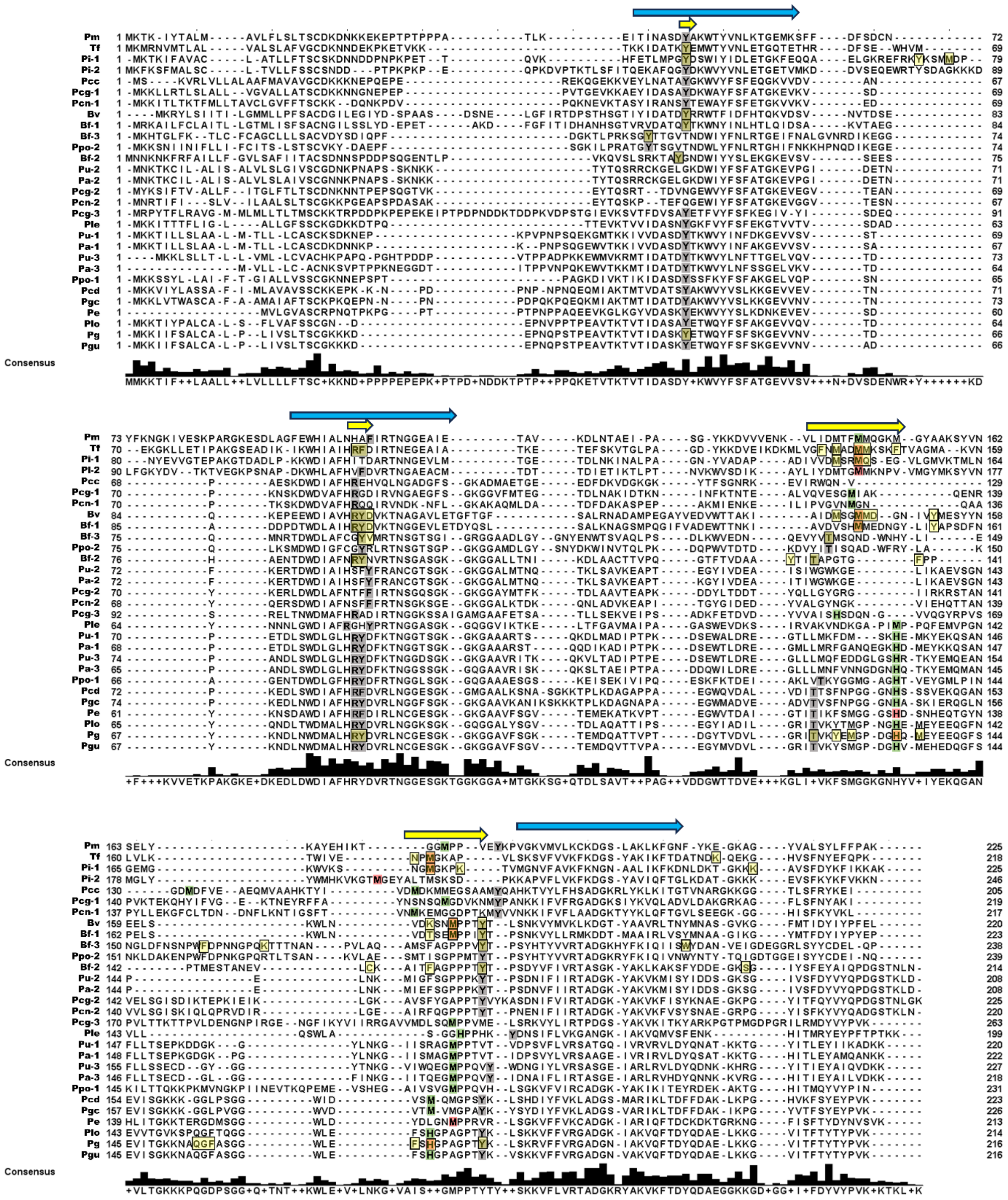

Fig. S2. Amino acid sequence alignment of HmuY proteins. Yellow arrows indicate regions of proteins with amino acids engaged in heme/PPIX binding. Blue arrows indicate protein regions that form the core of the protein structure. Amino acids involved in heme-iron coordination are shadowed in red (experimentally confirmed) or green (predicted). Amino acids forming ligand-binding pockets in proteins with resolved structures are shown in yellow with black edges. Predicted amino acids potentially involved in the PPIX ring binding are shown in grey. The consensus amino acid sequence is shown below the examined sequences. Species names with abbreviations, given along with HmuY names, are listed in Table 1 and Table S1.

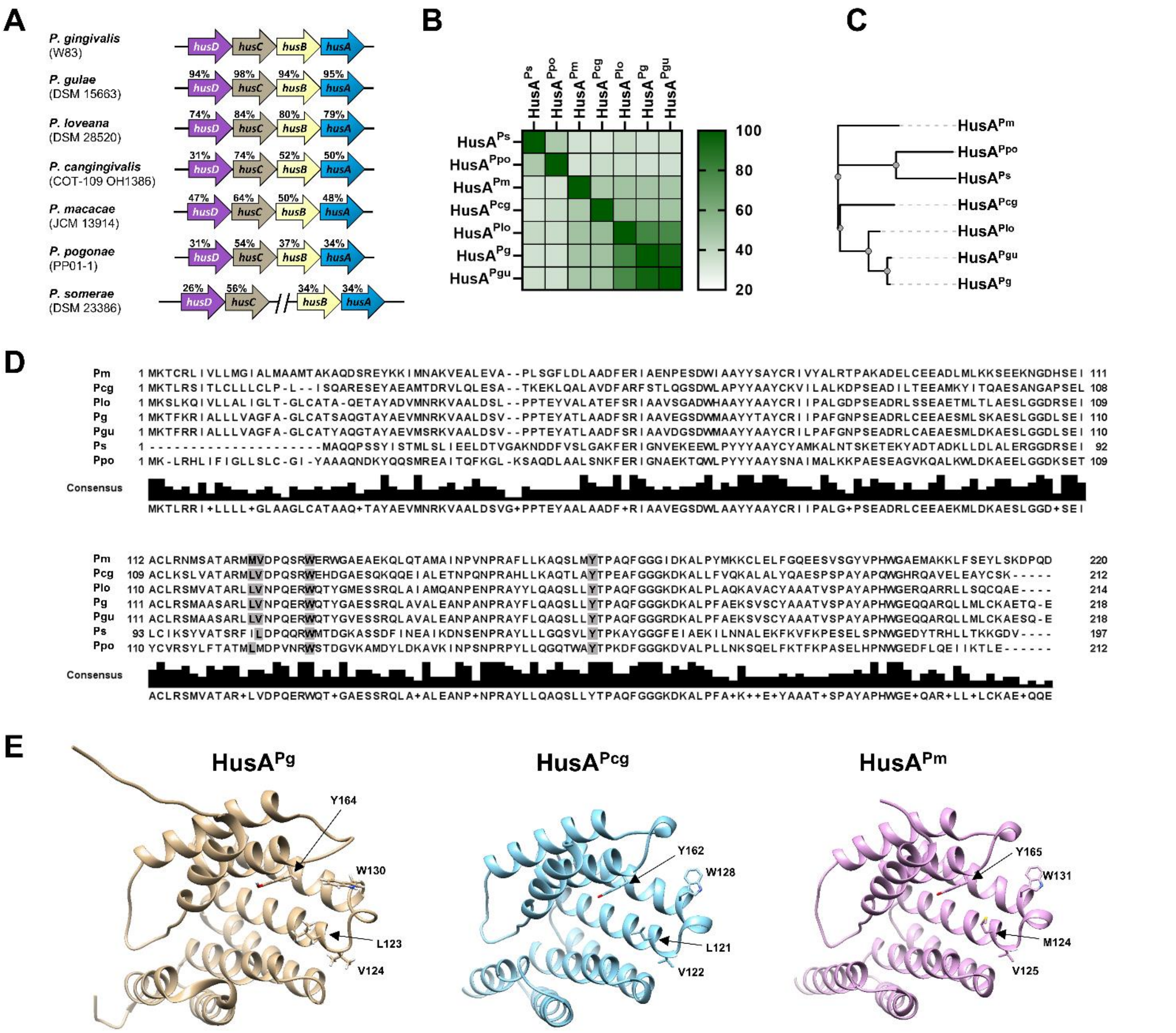

Fig. S3. Comparison of *Porphyromonas* HusA proteins. (A) Organization of the operons or gene clusters encoding proteins of the Hus system found in *Porphyromonas* species. A gap between genes marked by / / indicates that the gene clusters are in different genome regions. The values above the genes indicate the identity of the amino acid sequences concerning the protein from *Porphyromonas gingivalis*. Strain names are shown in brackets. (B) Heat map constructed based on amino acid sequence of HusA proteins. The color gradient from white to dark green shows the percentage of identity from lowest (20%) to highest (100%). (C) The phylogenetic tree was constructed based on the amino acid sequence of HusA proteins. (D) Amino acid sequence alignment of HusA proteins. Amino acids potentially involved in heme/PPIX binding are shadowed. The consensus amino acid sequence is shown below the examined sequences. Species names with abbreviations, given along with HusA names, are listed in Table 1 and Table S1. (E) Experimentally solved structure of HusA protein from *P. gingivalis* ($HusA^{Pg}$; PDB ID: 6CRL) and modeled structures of HusA homologs from *Porphyromonas cangingivalis* ($HusA^{Pcg}$) and *Porphyromonas macacae* ($HusA^{Pm}$) with localization of amino acids potentially involved in heme/PPIX binding. The structures of HusA proteins were modeled with AlphaFold (https://alphafold.com) (Jumper et al. 2021; Varadi et al. 2022). Protein structures were visualized with UCSF Chimera (https://www.cgl.ucsf.edu/chimera/) (Pettersen et al. 2004).

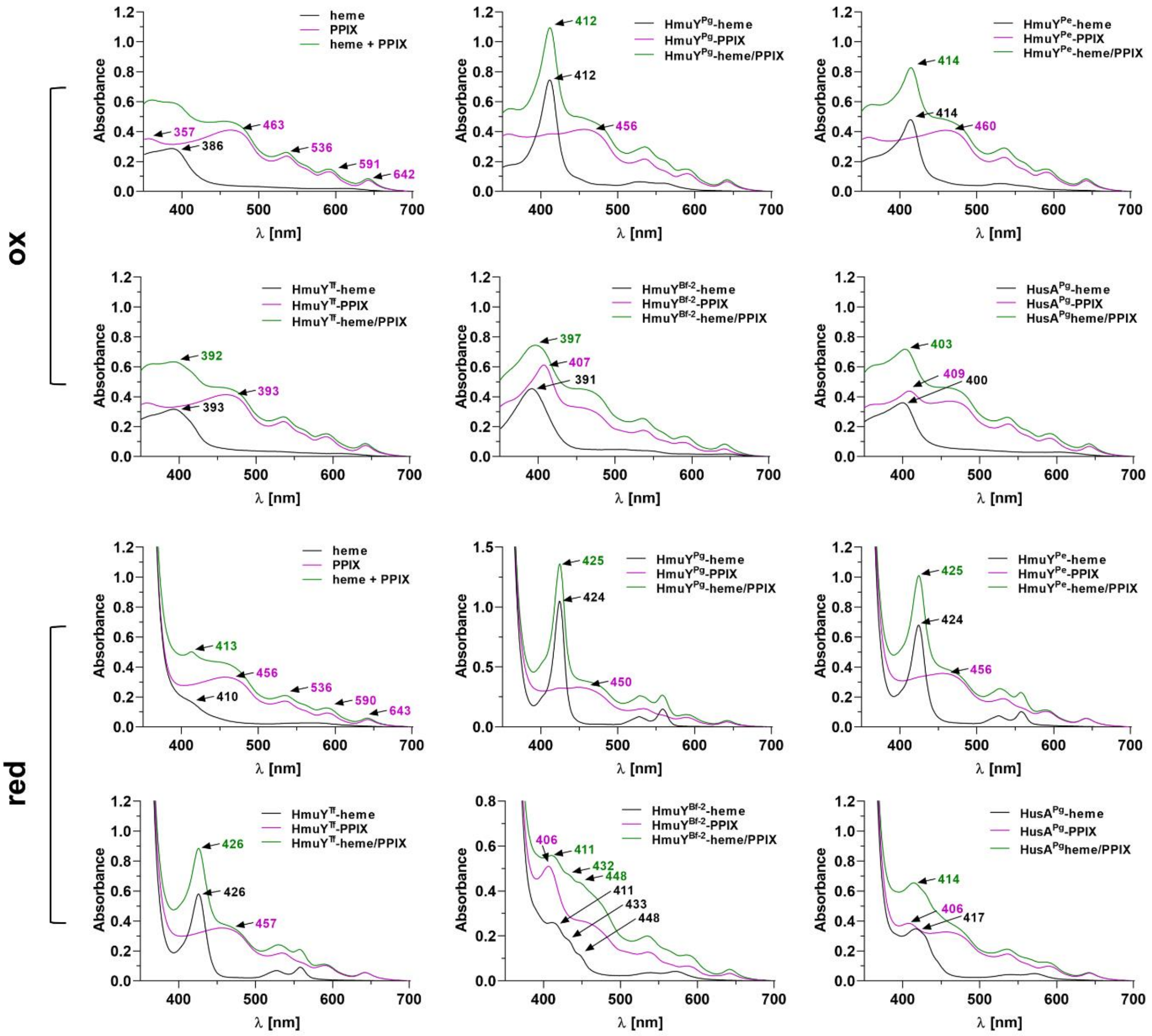

Fig. S4. Heme- and PPIX-binding capacity. *Porphyromonas gingivalis* HmuY^{Pg}, *Porphyromonas endodontalis* HmuY^{Pe}, and *Tannerella forsythia* HmuY^{Tf}, or *Bacteroides fragilis* HmuY^{Bf-2} and *P. gingivalis* HusA^{Pg} proteins were selected to demonstrate differences in heme alone (black lines), PPIX alone (purple lines), or heme in the presence of PPIX (green lines) binding resulting from the presence of two histidines (HmuY^{Pg}), a histidine-methionine pair (HmuY^{Pe}), two methionines (HmuY^{Tf}), or the absence of amino acids coordinating heme-iron (HmuY^{Bf-2} and HusA^{Pg}). The binding was examined using UV-visible absorbance spectroscopy. Spectra were recorded under oxidizing (ox) and reducing (red) conditions, the latter conditions formed by 10 mM sodium dithionite. Spectra of heme, PPIX, or a mixture of both porphyrin compounds are shown to demonstrate differences between porphyrins alone and protein-porphyrin complexes.

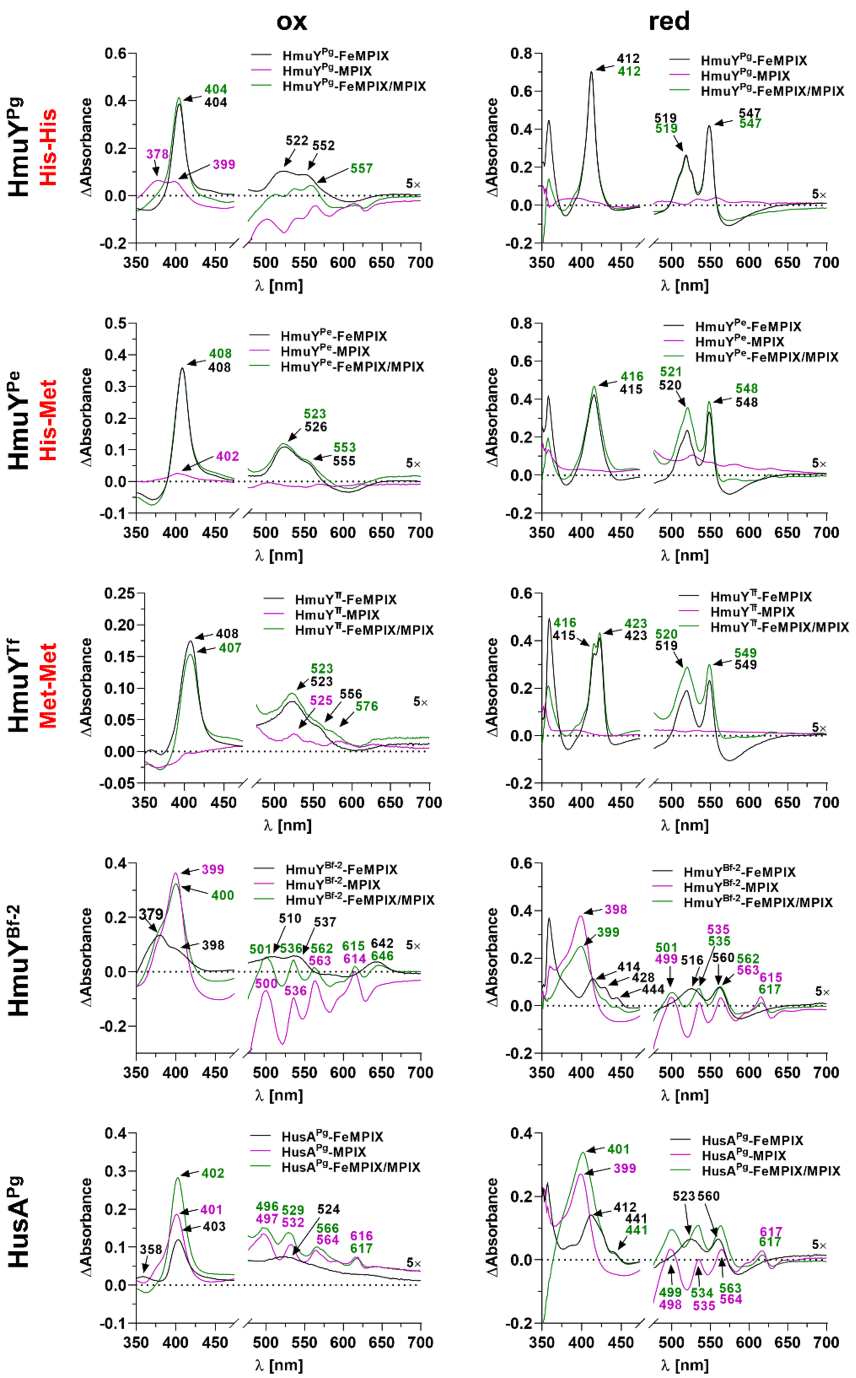

Fig. S5. FeMPIX- and MPIX-binding capacity. *Porphyromonas gingivalis* HmuY^{Pg}, *Porphyromonas endodontalis* HmuY^{Pe}, and *Tannerella forsythia* HmuY^{Tf}, or *Bacteroides fragilis* HmuY$^{Bf\text{-}2}$ and *P. gingivalis* HusA^{Pg} proteins were selected to demonstrate differences in FeMPIX alone (black lines), MPIX alone (purple lines) or FeMPIX in the presence of MPIX (green lines) binding resulting from the presence of two histidines (HmuY^{Pg}), a histidine-methionine pair (HmuY^{Pe}), two methionines (HmuY^{Tf}), or the absence of amino acids coordinating heme-iron (HmuY$^{Bf\text{-}2}$ and HusA^{Pg}). The binding was examined using difference absorbance spectroscopy (ΔAbsorbance). Spectra were recorded under oxidizing (ox) and reducing (red) conditions, the latter conditions formed by 10 mM sodium dithionite.

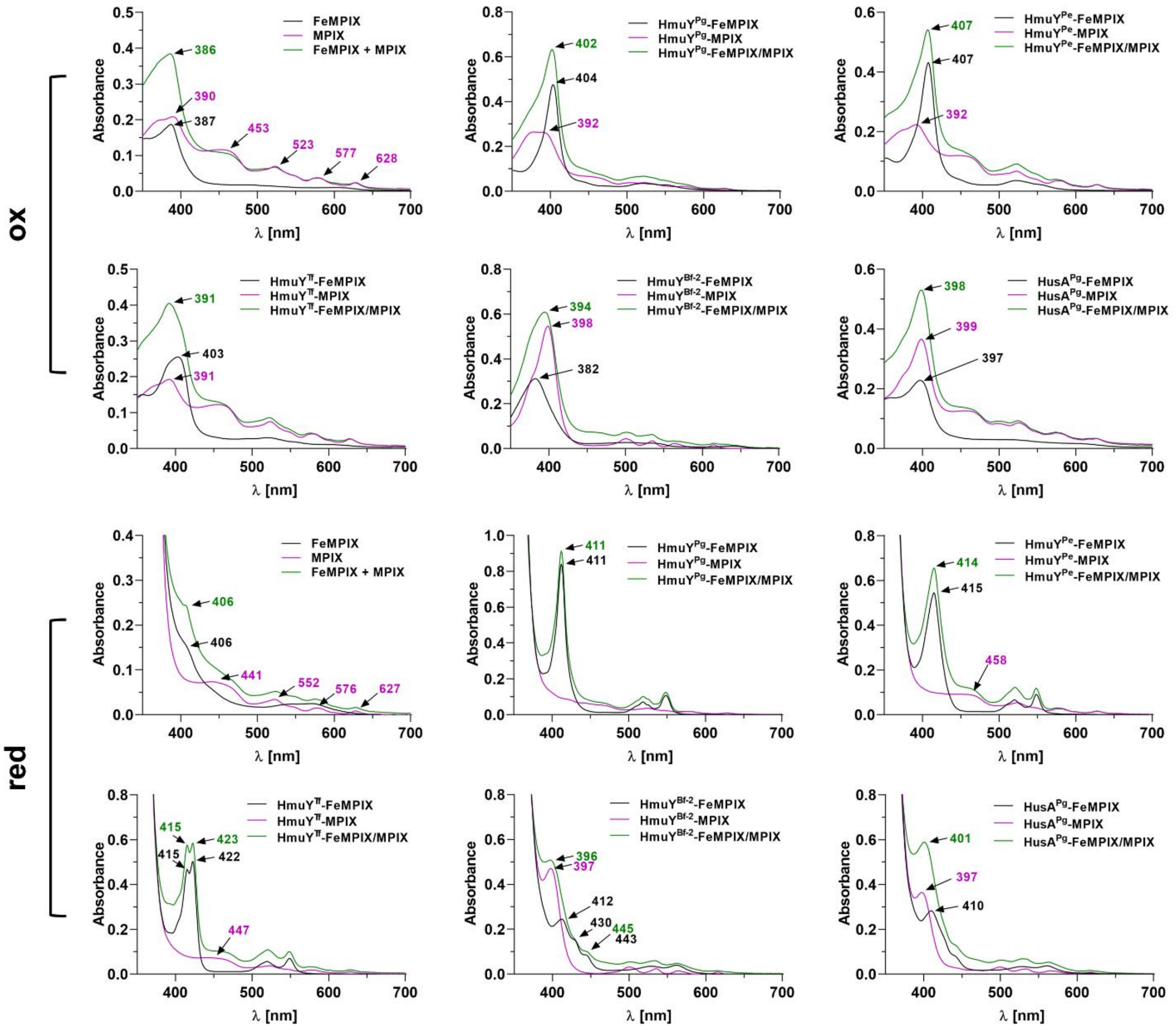

Fig. S6. FeMPIX- and MPIX-binding capacity. *Porphyromonas gingivalis* (HmuY^{Pg}), *Porphyromonas endodontalis* (HmuY^{Pe}), and *Tannerella forsythia* (HmuY^{Tf}), or *Bacteroides fragilis* (HmuY$^{Bf\text{-}2}$) and *P. gingivalis* HusA (HusA^{Pg}) proteins were selected to demonstrate differences in FeMPIX alone (black lines), MPIX alone (purple lines) or FeMPIX in the presence of MPIX (green lines) binding resulting from the presence of two histidines (HmuY^{Pg}), a histidine-methionine pair (HmuY^{Pe}), two methionines (HmuY^{Tf}), or the absence of amino acids coordinating heme-iron (HmuY$^{Bf\text{-}2}$ and HusA^{Pg}). The binding was examined using UV-visible absorbance spectroscopy. Spectra were recorded under oxidizing (ox) and reducing (red) conditions, the latter conditions formed by 10 mM sodium dithionite. Spectra of FeMPIX, MPIX, or a mixture of both porphyrin compounds are shown to demonstrate differences between porphyrins alone and protein-porphyrin complexes.

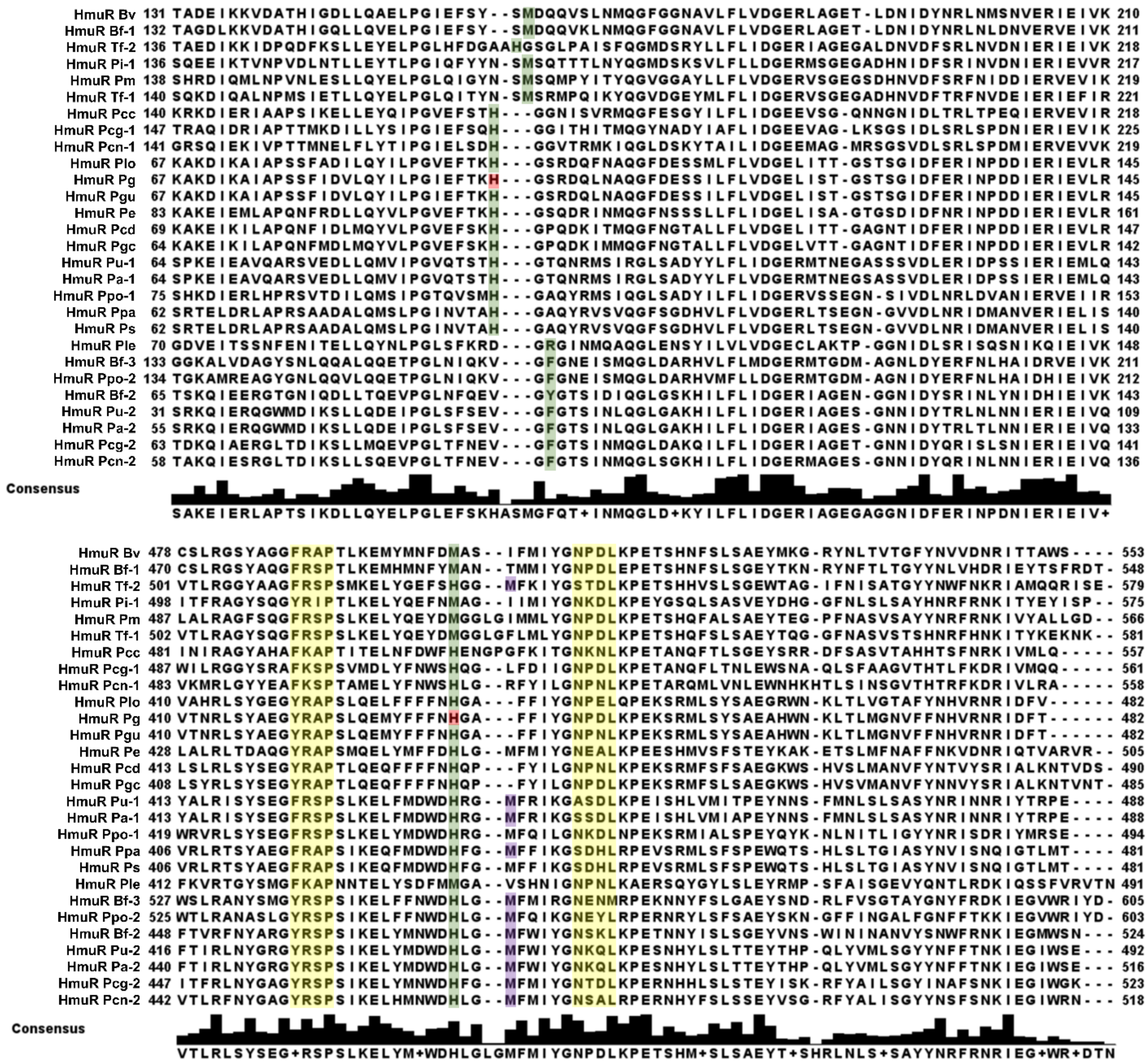

Fig. S7. Comparative analysis of HmuR TonB-dependent receptors (TDRs) of *Porphyromonas* species and other representative members of the Bacteroidota phylum. Amino acid sequence alignment of the selected regions of HmuR proteins is shown. Conserved amino acid motifs are shadowed in yellow, conserved heme-binding histidines, experimentally confirmed in the *Porphyromonas gingivalis* HmuR, are shadowed in red, and their predicted counterparts in other HmuR proteins are shadowed in green or purple. The consensus amino acid sequence is shown below the compared sequences. Species names with abbreviations, given along with HmuR, are listed in Table 1 and Table S1.

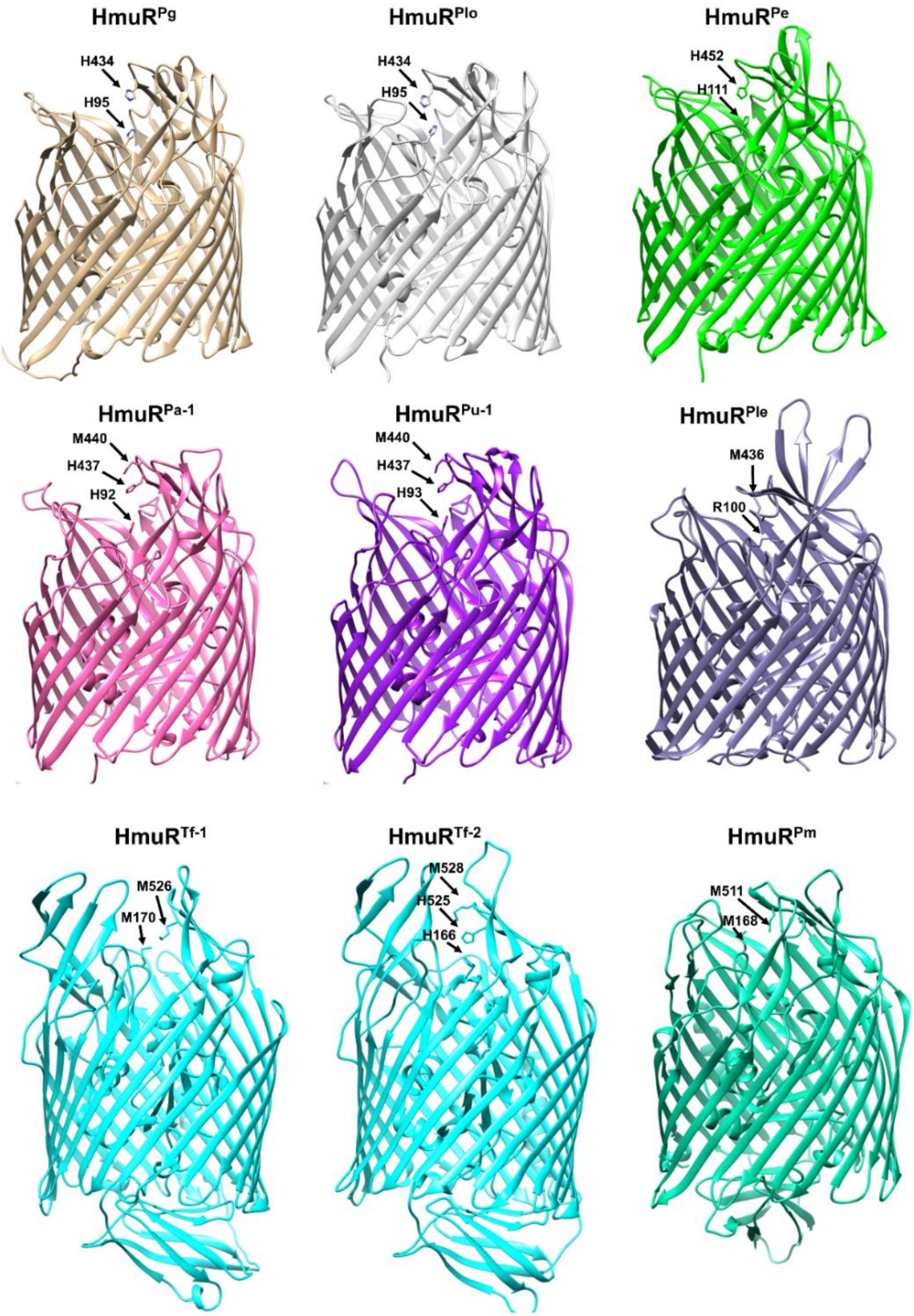

Fig. S8. Comparison of overall three-dimensional structures of HmuR proteins identified in representative *Porphyromonas* species and *Tannerella forsythia* (HmuR^{Tf-1} and HmuR^{Tf-2}). The positions of amino acids engaged in heme binding determined experimentally in HmuR^{Pg} or predicted in other HmuR proteins are marked with arrows. The structures of HmuR proteins were modeled with AlphaFold (https://alphafold.com) (Jumper et al. 2021; Varadi et al. 2022). Protein structures were visualized with UCSF Chimera (https://www.cgl.ucsf.edu/chimera/) (Pettersen et al. 2004). Abbreviations of species names, given along with HmuR, are listed in Table 1 and Table S1.

Table S1. Accession numbers of protein sequences and IDs of three-dimensional structures of HmuY, HmuR, and HusA proteins identified in *Porphyromonas* species and other representative members of the Bacteroidota phylum.

**Species name**	**Protein name**	**Protein sequence (NCBI accession number)**	**Source of three-dimensional protein structure (ID)**
Porphyromonas gingivalis	HmuY^{Pg}	AKV63662.1	PDB (3H8T; 6EWM)
	HmuR^{Pg}	AKV63661.1	AlphaFold (AF-Q7MUG9-F1)
	HusA^{Pg}	AKV65255.1	PDB (6CRL)
Porphyromonas gulae	HmuY^{Pgu}	WP_039438181.1	AlphaFold (AF-A0A099WTH2-F1)
	HmuR^{Pgu}	WP_018964856.1	AlphaFold (AF-A0A0A2EWH6-F1)
	HusA^{Pgu}	WP_018965124.1	
Porphyromonas loveana	HmuY^{Plo}	WP_116679143.1	AlphaFold (AF-A0A2U1FHF1-F1)
	HmuR^{Plo}	WP_116679142.1	AlphaFold (AF-A0A2U1FHG6-F1)
	HusA^{Plo}	WP_116679466.1	
Porphyromonas endodontalis	HmuY^{Pe}	EEN82265.1	AlphaFold (AF-C3JC46-F1)
	HmuR^{Pe}	WP_004334615.1	AlphaFold (AF-C3JC45-F1)
Porphyromonas circumdentaria	HmuY^{Pcd}	WP_078737171.1	AlphaFold (AF-A0A1T4NY32-F1)
	HmuR^{Pcd}	WP_078737170.1	AlphaFold (AF-A0A1T4NXX1-F1)
Porphyromonas gingivicanis	HmuY^{Pgc}	WP_025843422.1	AlphaFold (AF-A0A0A2GAC0-F1)
	HmuR^{Pgc}	WP_262498461.1	Phyre2[a,b]; I-TASSER[1]
Porphyromonas uenonis	HmuY^{Pu-1}	WP_007365951.1	AlphaFold (AF-C2MDL4-F1)
	HmuY^{Pu-2}	WP_007365011.1	AlphaFold (AF-C2MAS7-F1)
	HmuY^{Pu-3}	WP_007364618.1	AlphaFold (AF-C2M9P3-F1)
	HmuR^{Pu-1}	WP_007365947.1	AlphaFold (AF-C2MDL3-F1)
	HmuR^{Pu-2}	WP_232501405.1	AlphaFold (AF-C2MAS6-F1)
Porphyromonas asaccharolytica	HmuY^{Pa-1}	WP_013760568.1	AlphaFold (AF-F4KLQ3-F1)
	HmuY^{Pa-2}	WP_004330373.1	AlphaFold (AF-F4KMV2-F1)
	HmuY^{Pa-3}	WP_245528052.1	AlphaFold (AF-F4KJB9-F1)
	HmuR^{Pa-1}	WP_013760567.1	AlphaFold (AF-F4KLQ2-F1)
	HmuR^{Pa-2}	WP_013760707.1	AlphaFold (AF-F4KMV1-F1)
Porphyromonas levii	HmuY^{Ple}	WP_018358608.1	AlphaFold (AF-A0A4Y8WQ16-F1)
	HmuR^{Ple}	WP_018358607.1	AlphaFold (AF-A0A4Y8WQ48-F1)
Porphyromonas cangingivalis	HmuY^{Pcg-1}	WP_078735519.1	AlphaFold (AF-A0A1T4JSJ8-F1)
	HmuY^{Pcg-2}	WP_025837250.1	AlphaFold (AF-A0A0A2EPN1-F1)
	HmuY^{Pcg-3}	WP_036854131.1	AlphaFold (AF-A0A1T4NRN8-F1)
	HmuR^{Pcg-1}	WP_078735518.1	AlphaFold (AF-A0A1T4JSK0-F1)
	HmuR^{Pcg-2}	WP_051522656.1	AlphaFold (AF-A0A1T4JZ82-F1)
	HusA^{Pcg}	WP_036851981.1	AlphaFold (AF-A0A0A2EQD9-F1)
Porphyromonas canoris	HmuY^{Pcn-1}	WP_052089871.1	Phyre2[c,d]; I-TASSER[2]
	HmuY^{Pcn-2}	WP_036788494.1	Phyre2[c,d]; I-TASSER[2]
	HmuR^{Pcn-1}	WP_081964512.1	Phyre2[e,f]; I-TASSER[3]
	HmuR^{Pcn-2}	WP_036788496.1	Phyre2[a,e]; I-TASSER[3]
Porphyromonas macacae	HmuY^{Pm}	WP_018360193.1	AlphaFold (AF-A0A379E6Z1-F1)
	HmuR^{Pm}	WP_018360192.1	AlphaFold (AF-A0A379DHE0-F1)
	HusA^{Pm}	WP_018360786.1	AlphaFold (AF-A0A379DK08-F1)
Porphyromonas pasteri	HmuR^{Ppa}	WP_188807413.1	Phyre2[b,e]; I-TASSER[3]
Porphyromonas somerae	HmuR^{Ps}	WP_060935907.1	AlphaFold (AF-A0A134B229-F1)
	HusA^{Ps}	WP_051052328.1	
Porphyromonas crevioricanis	HmuY^{Pcc}	WP_023939427.1	AlphaFold (AF-A0A0A2FIS2-F1)
	HmuR^{Pcc}	WP_023939425.1	AlphaFold (AF-A0A2X4STT9-F1)
Porphyromonas pogonae	HmuY^{Ppo-1}	WP_329903936.1	Phyre2[c,d]; I-TASSER[4]
	HmuY^{Ppo-2}	WP_329902994.1	Phyre2[g,d]; I-TASSER[5]
	HmuR^{Ppo-1}	WP_329903938.1	Phyre2[h,a]; I-TASSER[6]
	HmuR^{Ppo-2}	WP_329902993.1	Phyre2[b,f]; I-TASSER[7]
	HusA^{Ppo}	WP_329904683.1	
Tannerella forsythia	HmuY^{Tf} (Tfo)	WP_014225394.1	PDB (6EU8)
	HmuR^{Tf-1}	WP_014225393.1	AlphaFold (AF-G8UQX5-F1)

	$HmuR^{Tf-2}$	WP_208854689.1	AlphaFold (AF-G8UQX4-F1)
Prevotella intermedia	$HmuY^{Pi-1}$ (PinO)	WP_014708291.1	PDB (6R2H)
	$HmuY^{Pi-2}$ (PinA)	WP_014709321.1	AlphaFold (AF-A0A1P8JJ12-F1)
	$HmuR^{Pi}$	WP_044047471.1	AlphaFold (AF-A0A1P8JPE5-F1)
Bacteroides fragilis	$HmuY^{Bf-1}$ (BfrA)	WP_010993105.1	PDB (4GBS)
	$HmuY^{Bf-2}$ (BfrB)	WP_005785524.1	PDB (8B6A)
	$HmuY^{Bf-3}$ (BfrC)	WP_005787358.1	PDB (8B61)
	$HmuR^{Bf-1}$	WP_005815393.1	AlphaFold (AF-Q5LBW4-F1)
	$HmuR^{Bf-2}$	WP_005793172.1	AlphaFold (AF-E1WQ73-F1)
	$HmuR^{Bf-3}$	WP_010992887.1	AlphaFold (AF-A0A642L6Q0-F1)
Bacteroides vulgatus	$HmuY^{Bv}$ (Bvu)	WP_005839056.1	PDB (3U22)
	$HmuR^{Bv}$	WP_005848805.1	AlphaFold (AF-A6L2E0-F1)

[a]modeled based on the structure of siderophore receptor PirA from *Acinetobacter baumannii* (PDB ID: 5FR8)
[b]modeled based on the structure of the colicin I receptor Cir from *Escherichia coli* (PDB ID: 2HDI)
[c]modeled based on the $HmuY^{Pg}$ structure (PDB ID: 3H8T)
[d]modeled based on the $HmuY^{Bf-2}$ (formerly BfrB) structure (PDB ID: 8B6A)
[e]modeled based on the structure of ferric enterobactin receptor (PfeA) from *Pseudomonas aeruginosa* (PDB ID: 5MZS)
[f]modeled based on the structure of substrate-free levan utilization machinery *Bacteroides thetaiotaomicron* (PDB ID: 8A9Y)
[g]modeled based on the $HmuY^{Bf-3}$ (formerly BfrC) structure (PDB ID: 8B61)
[h]modeled based on the structure of SusC components of the dextran utilization system from *Bacteroides thetaiotaomicron* (PDB ID: 8AA4)
[1]the highest structural analogy is shown by I-TASSER to ferric pyoverdine outer membrane receptor FpvA from *Pseudomonas aeruginosa* (PDB ID: 2O5P), ferrioxamine B transporter FoxA from *Pseudomonas aeruginosa* (PDB ID: 6I96)
[2]the highest structural analogy is shown by I-TASSER to the $HmuY^{Pg}$ (PDB ID: 3H8T), $HmuY^{Tf}$ (formerly) (PDB ID: 6EU8)
[3]the highest structural analogy is shown by I-TASSER to ferric pyoverdine outer membrane receptor FpvA from *Pseudomonas aeruginosa* (PDB ID: 2O5P), the structure of ferric enterobactin receptor (PfeA) from *Pseudomonas aeruginosa* (PDB ID: 5MZS)
[4]the highest structural analogy is shown by I-TASSER to the $HmuY^{Pg}$ (PDB ID: 3H8T), BfrB (PDB ID: 8B6A),
[5]the highest structural analogy is shown by I-TASSER to the $HmuY^{Bf-3}$ (formerly BfrC) (PDB ID: 8B61), $HmuY^{Pg}$ (PDB ID: 3H8T)
[6]the highest structural analogy is shown by I-TASSER to the structure of ferric enterobactin receptor (PfeA) from *Pseudomonas aeruginosa* (PDB ID: 5MZS), ferrioxamine B transporter FoxA from *Pseudomonas aeruginosa* (PDB ID: 6I96).
[7]the highest structural analogy is shown by I-TASSER to the structure of the ferric enterobactin receptor (PfeA) from *Pseudomonas aeruginosa* (PDB ID: 5MZS), the ferripyoverdine receptor (FpvA) from *Pseudomonas aeruginosa* (PDB ID: 2W78).

Table S2. Plasmids generated and/or used in this study.

Plasmid name	Description*	Reference
pMAL-c5x_His-HmuY	plasmid used to overexpress $HmuY^{Pg}$ protein with 6×His-MBP** tag at the N-terminus	Śmiga et al. 2023
pMAL-c5x_His-HmuY-H134M	modified pMAL-c5x_His-HmuY plasmid used to overexpress $HmuY^{Pg}$ H134M protein variant with 6×His-MBP tag at the N-terminus	Kosno et al. 2022
pMAL-c5x_His-HmuY-H166M	modified pMAL-c5x_His-HmuY plasmid used to overexpress $HmuY^{Pg}$ H166M protein variant with 6×His-MBP tag at the N-terminus	Kosno et al. 2022
pMAL-c5x_His-HmuY-H134M/H166M	modified pMAL-c5x_His-HmuY plasmid used to overexpress $HmuY^{Pg}$ H134M/H166M protein variant with 6×His-MBP tag at the N-terminus	Kosno et al. 2022
pMAL-c5x_His-HusA	plasmid used to overexpress $HusA^{Pg}$ protein with a 6×His-MBP tag at the N-terminus	Śmiga et al. 2023
pTriEx-$HmuY^{Pe}$	plasmid used to overexpress $HmuY^{Pe}$ protein with 6×His at the protein N-terminus	Śmiga and Olczak 2024
pTriEx-$HmuY^{Pe}$H128M	modified pTriEx-$HmuY^{Pe}$ plasmid used to overexpress $HmuY^{Pe}$ H128M protein variant with 6×His at the N-terminus	This study
pTriEx-$HmuY^{Pe}$M163H	modified pTriEx-$HmuY^{Pe}$ plasmid used to overexpress $HmuY^{Pe}$ M163 protein variant with 6×His at the N-terminus	This study
pTriEx-$HmuY^{Pe}$H128M/M163H	modified pTriEx-$HmuY^{Pe}$ plasmid used to overexpress $HmuY^{Pe}$ H128M/M163H protein variant with 6×His at the N-terminus	This study
pMALc5x_Tfo	plasmid used to overexpress $HmuY^{Tf}$ protein with 6×His-MBP tag at the N-terminus	Ślęzak et al. 2020
pMALc5x_$HmuY^{Tf}$M145H	modified pMALc5x_Tfo plasmid used to overexpress $HmuY^{Tf}$ M145H protein variant with 6×His-MBP tag at the N-terminus	This study
pMALc5x_$HmuY^{Tf}$M171H	modified pMALc5x_Tfo plasmid used to overexpress $HmuY^{Tf}$ M171H protein variant with 6×His-MBP tag at the N-terminus	This study
pMALc5x_$HmuY^{Tf}$M145/M171H	modified pMALc5x_Tfo plasmid used to overexpress $HmuY^{Tf}$ M145H/M171H protein variant with 6×His-MBP tag at the N-terminus	This study
pMal-c5x_His-BfrB	plasmid used to overexpress $HmuY^{Bf-2}$ protein with 6×His-MBP tag at the N-terminus	Antonyuk et al. 2023
pMal-c5x_His-BfrB_Y89A	modified pMal-c5x_His-BfrB plasmid used to overexpress $HmuY^{Bf-2}$ Y89A protein variant with 6×His-MBP tag at the N-terminus	Antonyuk et al. 2023
pMal-c5x_His-BfrB_M144A	modified pMal-c5x_His-BfrB plasmid used to overexpress $HmuY^{Bf-2}$ M144A protein variant with 6×His-MBP tag at the N-terminus	Antonyuk et al. 2023
pMal-c5x_His-BfrB_C153A	modified pMal-c5x_His-BfrB plasmid used to overexpress $HmuY^{Bf-2}$ C153A protein variant with 6×His-MBP tag at the N-terminus	This study
pMal-c5x_His-BfrB_Y165A	modified pMal-c5x_His-BfrB plasmid used to overexpress $HmuY^{Bf-2}$ Y165A protein variant with 6×His-MBP tag at the N-terminus	Antonyuk et al. 2023

*All tags were removed from recombinant proteins during the purification process using Factor Xa.

**MBP – maltose binding protein

Table S3. Primers designed and used in this study. All primers were used to introduce point mutations into respective plasmids.

Primer name	DNA sequence 5`→3`	Description
PBM_0858	GCATGGGAGGTGGCTCTATGGATAGTAATCACG AGCAAACAGG	primers used to replace histidine 128 with methionine (H128M) in HmuY^{Pe} encoded in pTriEx-HmuY^{Pe} plasmid
PBM_0859	CCTGTTTGCTCGTGATTACTATCCATAGAGCCAC CTCCCATGC	
PBM_0860	TGATTACGACCTTGGCAACCACCCTCCCCGCGT TCGTTTGTC	primers used to replace methionine 163 with histidine (M163H) in HmuY^{Pe} encoded in pTriEx-HmuY^{Pe} plasmid
PBM_0861	AACGAACGCGGGGAGGGTGGTTGCCAAGGTCG TAATCAAGCC	
PBM_0862	GTTGGTCGGTTTCAATATGGCGGATCACATGAA AAGTAAATTTACTGTTGC	primers used to replace methionine 145 with histidine (M145H) in HmuY^{Tf} encoded in pMALc5x_Tfo plasmid
PBM_0863	GGCAACAGTAAATTTACTTTTCATGTGATCCGC CATATTGAAACCGACCAAC	
PBM_0864	CTCAAAACATGGATTGTGGAAAATCCTCACGGA AAGGCACCCGTACTCTCCAAATCC	primers used to replace methionine 171 with histidine (M171H) in HmuY^{Tf} encoded in pMALc5x_Tfo plasmid
PBM_0865	GGATTTGGAGAGTACGGGTGCCTTTCCGTGAGG ATTTTCCACAATCCATGTTTTGAG	
PBM_0685	AGTCCACCGCTAATGAGGTTCTCGCGAAAGCAA TCACTTTTGCCGGCCC	primers used to replace cysteine 153 with alanine (C153A) in HmuY^{Bf-2} encoded in pMal-c5x_His-BfrB_C153A plasmid
PBM_0686	GGGCCGGCAAAAGTGATTGCTTTCGCGAGAACC TCATTAGCGGTGGACT	

Table S4. Comparison of *Porphyromonas* species.

Species (abbreviation used in this study)	Strain	Accession number	Number of contigs	Genome size [Mb]	GC content [%]	Number of proteins	Host	Isolation source	Selected references
P. gingivalis (Pg)	W83	NZ_CP025932.1	1	2.3	48.5	1919	human	oral cavity	Summanen et al. 2015, Bird et al. 2016
P. gulae (Pgu)	DSM 15663	GCF_000378065.1	67	2.3	48,5	1948	mammals (first isolation from a dog)	oral cavity	Fournier et al. 2001, Summanen et al. 2015, Bird et al. 2016
P. loveana (Plo)	DSM 28520	GCF_003096695.1	45	2.3	50.0	1826	marsupials	oral cavity	Bird et al.2016
P. endodontalis (Pe)	ATCC 35406	GCF_000174815.1	37	2.1	47.5	1965	human	oral cavity	Summanen et al. 2015, Bird et al. 2016
P. circumdentaria (Pcd)	ATCC 51356	GCF_900167105.1	39	2.0	43.0	1725	feline	oral cavity, empyemas	Summanen et al. 2015, Bird et al. 2016
P. gingivicanis (Pgc)	JCM 15907	GCF_000614585.1	35	2.1	42.5	1657	dog	oral cavity	Summanen et al. 2015, Bird et al. 2016, Hirasawa and Takada 1994
P. creviorícanis (Pcc)	JCM 13913	GCF_000509265.1	89	2.1	45.5	1710	dog	oral cavity	Sakamoto and Ohkuma 2013, Summanen et al. 2015, Bird et al. 2016, Hirasawa and Takada 1994
P. levii (Ple)	DSM 23370	GCF_000379925.1	125	2.5	45.5	2066	bovine	rumen, abscess	Summanen et al. 2015, Bird et al. 2016
P. uenonis (Pu)	60-3	GCF_000174775.1	250	2.2	52.5	1806	human	gut	Finegold et al. 2004, Summanen et al. 2015, Bird et al. 2016
P. asaccharolytica (Pa)	DSM 20707	NC_015501.1	1	2.2	52.5	1644	human	empyema	Summanen et al. 2015, Bird et al. 2016
P. cangingivalis (Pcg)	JCM 15983	GCF_000614355.1	48	2.4	47.5	1896	dog	oral cavity	Collins et al. 1994, Summanen et al. 2015, Bird et al. 2016
P. canoris (Pcn)	COT-108 OH1224	GCF_000765975.1	21	2.3	44.5	1846	dog	oral cavity	Love et al. 1994, Summanen et al. 2015, Bird et al. 2016
P. macacae (Pm)	JCM 13914	GCF_000614325.1	44	2.2	43.5	1685	feline and monkeys	oral cavity	Love 1995, Summanen et al. 2015, Bird et al. 2016
P. pogonae (Ppo)	PP01-1	NZ_CP143258.1	1	2.9	41.5	2223	lizard	abscess acute sinusitis,	Kawamura et al. 2015, Kim et al. 2016, Huang et al. 2024

							human	infected wound	
P. pasteri (Ppa)	JCM 30531	GCF_014647755.1	9	2.2	56.0	1626	human	oral cavity	Sakamoto et al. 2015
P. somerae (Ps)	DSM 23386	GCF_000372405.1	95	2.4	47.0	1881	human	ulcer	Summanen et al. 2005, Summanen et al. 2015, Bird et al. 2016
P. bennonis	JCM 16335	GCF_000375645.1	87	2.0	56.5	1571	human	abscess	Summanen et al. 2009, Summanen et al. 2015, Bird et al. 2016,
P. catoniae	ATCC 51270	GCF_000565015.1	25	2.0	51.0	1561	human	oral cavity	Summanen et al. 2015, Bird et al. 2016
P. bronchialis	PAGU1601	No genome sequence available. Closely related to *P. catoniae* and *P. pogonae*					human	bronchial fluids	Sato et al. 2015
P. katsikii	JF5581	No genome sequence available. Closely related to *P. somerae* and *P. levii*					goat	lung	Filioussis et al. 2015

References

Antonyuk SV, Siemińska K, Śmiga M, Strange RW, Wagner M, Barnett KJ, Olczak T. 2023. *Bacteroides fragilis* expresses three proteins similar to *Porphyromonas gingivalis* HmuY: hemophore-like proteins differentially evolved to participate in heme acquisition in oral and gut microbiomes. *FASEB J*. 37(7):e22981. doi: 10.1096/fj.202300366R.

Bird PS, Trott DJ, Mikkelsen D, Milinovich GJ, Hillman KM, Burrell PC, Blackall LL. 2016. *Porphyromonas loveana* sp. nov., isolated from the oral cavity of Australian marsupials. *Int J Syst Evol Microbiol*. 66:3771-3778. doi: 10.1099/ijsem.0.000898.

Collins MD, Love DN, Karjalainen J, Kanervo A, Forsblom B, Willems A, Stubbs S, Sarkiala E, Bailey GD, Wigney DI, et al. 1994. Phylogenetic analysis of members of the genus *Porphyromonas* and description of *Porphyromonas cangingivalis* sp. nov. and *Porphyromonas cansulci* sp. nov. *Int J Syst Bacteriol*. 44:674-679. doi: 10.1099/00207713-44-4-674.

Filioussis G, Petridou E, Karavanis E, Frey J. 2015. *Pyogranulomatous pneumonia* in goats caused by an undescribed *Porphyromonas* species, "*Porphyromonas katsikii*". *J Clin Microbiol*. 53(3):795-8. doi: 10.1128/JCM.02682-14.

Finegold SM, Vaisanen ML, Rautio M, Eerola E, Summanen P, Molitoris D, Song Y, Liu C, Jousimies-Somer H. 2004. *Porphyromonas uenonis* sp. nov., a pathogen for humans distinct from *P. asaccharolytica* and *P. endodontalis*. *J Clin Microbiol*. 42:5298-5301. doi: 10.1128/JCM.42.11.5298-5301.2004.

Fournier D, Mouton C, Lapierre P, Kato T, Okuda K, Menard C. 2001. *Porphyromonas gulae* sp. nov., an anaerobic, gram-negative coccobacillus from the gingival sulcus of various animal hosts. *Int J Syst Evol Microbiol*. 51(Pt3):1179-1189. doi: 10.1099/00207713-51-3-1179.

Hirasawa M, Takada K. 1994. *Porphyromonas gingivicanis* sp. nov. and *Porphyromonas crevioricanis* sp. nov., isolated from Beagles. *Int J Sys Bacteriol*. 44:637-640. doi: 10.1099/00207713-44-4-637.

Huang J, Zhu Y, Gong D, Ma S, Wu C. 2024. Whole genome sequence of *Porphyromonas pogonae* PP01-1, a human strain harboring *$bla_{OXA-347}$* and *tet(Q)* with chromosomal location. *J Glob Antimicrob Resist.* doi: 10.1016/j.jgar.2024.04.015.

Jumper J, Evans R, Pritzel A, Green T, Figurnov M, Ronneberger O, Tunyasuvunakool K, Bates R, Zidek A, Potapenko A, et al. 2021. Highly accurate protein structure prediction with AlphaFold. *Nature*. 596:583–589. doi: 10.1038/s41586-021-03819-2.

Kawamura Y, Kuwabara S, Kania SA, Kato, Hamagishi M, Fujiwara N, Sato T, Tomida J, Tanaka K, Bemis D. 2015. *Porphyromonas pogonae* sp. nov., an anaerobic but low concentration oxygen adapted coccobacillus isolated from lizards (*Pogona vitticeps*) or human clinical specimens, and emended description of the genus *Porphyromonas* Shah and Collins 1988. *Syst Appl Microbiol*. 38:104-109. doi: 10.1016/j.syapm.2014.11.004.

Kosno J, Sieminska K, Olczak T. 2022. Unique properties of heme binding of the *Porphyromonas gingivalis* HmuY hemophore-like protein result from the evolutionary adaptation of the protein structure. *Molecules*. 27(5):1703. doi: 10.3390/molecules27051703.

Kim B, Pai H, Hwang KT, Lee Y. 2016. *Porphyromonas pogonae* identification from a soft tissue infection: The first human case. *Anaerobe*. 42:37-39. doi: 10.1016/j.anaerobe.2016.08.002.

Love DN. 1995. *Porphyromonas macacae* comb. nov., a consequence of *Bacteroides macacae* being a senior synonym of *Porphyromonas salivosa*. *Int J Syst Bacteriol*. 45:90-92. doi: 10.1099/00207713-45-1-90.

Pettersen EF, Goddard TD, Huang CC, Couch GS, Greenblatt DM, Meng EC, Ferrin TE. 2004. UCSF Chimera--a visualization system for exploratory research and analysis. *J Comput Chem*. 5:1605-1612. doi: 10.1002/jcc.20084.

Sakamoto M, Li D, Shibata Y, Takeshita T, Yoshihisa Y, Ohkuma M. 2015. *Porphyromonas pasteri* sp. nov., isolated from human saliva. *Int J Syst Evol Microbiol*. 65:2511-2515. doi: 10.1099/ijs.0.000294.

Sakamoto M, Ohkuma M. 2013. *Porphyromonas crevioricanis* is an earlier heterotypic synonym of *Porphyromonas cansulci* and has priority. *Int J Syst Evol Microbiol*. 63(Pt 2):454-457. doi: 10.1099/ijs.0.042531-0.

Sato T, Tomida J, Naka T, Fujiwara N, Hasegawa A, Hoshikawa Y, Matsuyama J, Ishida N, Kondo T, Tanaka K, et al. 2015. *Porphyromonas bronchialis* sp. nov. isolated from intraoperative bronchial fluids of a patient with non-small cell lung cancer. *Tohoku J Exp Med*. 237:31-37. doi: 10.1620/tjem.237.31.

Ślęzak P, Śmiga M, Smalley JW, Siemińska K, Olczak T. 2020. *Porphyromonas gingivalis* HmuY and *Streptococcus gordonii* GAPDH—novel heme acquisition strategy in the oral microbiome. *Int J Mol Sci*. 21:4150. doi: 10.3390/ijms21114150.

Śmiga M, Olczak T. 2024. *Porphyromonas endodontalis* HmuY differentially participates in heme acquisition compared to the *Porphyromonas gingivalis* and *Tannerella forsythia* hemophore-like proteins. *Front Cell Infect Microbiol*. 14:1421018. doi: 10.3389/fcimb.2024.1421018.

Śmiga M, Ślęzak P, Wagner M, Olczak T. 2023. Interplay between *Porphyromonas gingivalis* hemophore-like protein HmuY and Kgp/RgpA gingipains plays a superior role in heme supply. *Microbiol Spectr*. 11(2):e0459322. doi: 10.1128/spectrum.04593-22.

Summanen PH, Durmaz P, Vaisanen ML, Liu C, Molitoris D, Eerola E, Helander IM, Finegold SM. 2005. *Porphyromonas somerae* sp. nov., a pathogen isolated from humans and distinct from *Porphyromonas levii*. *J Clin Microbiol*. 43:4455-4459. doi.org/10.1128/jcm.43.9.4455-4459.2005.

Summanen P, Finegold SM, Summanen P, Finegold SM. 2015. Bergey's Manual of Systematics of Archaea and Bacteria 1–14; John Wiley & Sons, Ltd.: Hoboken, NJ, USA. doi: 10.1002/9781118960608.gbm00246.

Summanen PH, Lawson PA, Finegold SM. 2009. *Porphyromonas bennonis* sp. nov., isolated from human clinical specimens. *Int J Syst Evol Microbiol*. 59(Pt 7):1727-1732. doi: 10.1099/ijs.0.001909-0.

Varadi M, Anyango S, Deshpande M, Nair S, Natassia C, Yordanova G, Yuan D, Stroe O, Wood G, Laydon A, et al. 2022. AlphaFold Protein Structure Database: massively expanding the structural coverage of protein sequence space with high-accuracy models. *Nucleic Acids Res*. 50:D439–D444. doi:10.1093/nar/gkab1061.
